# Phlorizin in Apple Trees: From Horticultural Factors to Nutraceutical Applications

**DOI:** 10.3390/cimb48101035

**Published:** 2026-10-06

**Authors:** Maria Grazia Bonomo, Anna Caruso, Giuliana Bianco, Rosaria Cozzolino, Emanuela Lavanga, Raweh Abdallah, Giovanni Salzano, Carmela Saturnino

**Affiliations:** 1 Department of Health Sciences, University of Basilicata, Viale dell’ Ateneo Lucano 10, 85100 Potenza, Italy; mariagrazia.bonomo@unibas.it (M.G.B.); anna.caruso@unibas.it (A.C.); emanuela.lavanga@unibas.it (E.L.); giovanni.salzano@unibas.it (G.S.); carmela.saturnino@unibas.it (C.S.); 2 School of Nutraceutical Science—People’s University of the New Salerno Medical School (UNIPOSMS), 84100 Salerno, Italy; giuliana.bianco@unibas.it; 3 Department of Basic and Applied Sciences, University of Basilicata, Viale dell’ Ateneo Lucano 10, 85100 Potenza, Italy; 4 Institute of Food Science, National Research Council (ISA-CNR), Via Roma 64, 83100 Avellino, Italy; 5 Cardiovascular Sciences Department, Agostino Gemelli Foundation Polyclinic IRCCS Largo Agostino Gemelli, 21, 00168 Rome, Italy; prof.reweh@icloud.com or abdallah.raweh@unicatt.it; 6 Cardiology Unit, Catholic University of the Sacred Heart, Largo Francesco Vito, 1, 00168 Roma, Italy

**Keywords:** phlorizin, apple trees, nutraceuticals, properties, horticultural factors

## Abstract

Phlorizin is a naturally occurring dihydrochalcone predominantly found in apple trees (*Malus domestica*), where it is one of the species’ characteristic phenolic compounds. Its accumulation varies according to genotype, tissue type, developmental stage, environmental conditions, and cultivation practices. While these factors are fundamental for understanding phlorizin biosynthesis and distribution, growing attention has focused on its biological activities and potential health applications. This review provides an integrated overview of phlorizin, from its occurrence in apple plants to its emerging nutraceutical and biomedical relevance. Particular emphasis is placed on the molecular mechanisms underlying its antioxidant, anti-inflammatory, antimicrobial, antiviral, and metabolic effects. Phlorizin is best known for inhibiting sodium–glucose co-transporters (SGLT1 and SGLT2), a mechanism that inspired the development of synthetic SGLT inhibitors for metabolic diseases. Beyond glucose regulation, increasing evidence highlights its broader effects on lipid metabolism, gut microbiota modulation, neuroprotection, cardioprotection, hepatoprotection, anti-aging pathways, and tissue regeneration. Advances in extraction, purification, encapsulation, and delivery technologies are expanding its applications in functional foods, dietary supplements, and biomaterials. However, limited bioavailability and scarce clinical evidence remain key challenges. Overall, phlorizin represents a promising apple-derived phytochemical with significant potential for future health-oriented applications.

## 1. Introduction

In recent years, natural bioactive compounds have attracted increasing attention in food science and preventive medicine owing to their potential to promote human health and reduce the risk of chronic diseases. Consequently, plant-derived nutraceuticals have emerged as promising tools for the prevention and complementary management of metabolic, inflammatory, and degenerative disorders [1,2]. Phytochemicals are broadly classified into polyphenols, terpenoids such as carotenoids, phytosterols, alkaloids, and organosulfur compounds, all of which modulate key physiological processes associated with health maintenance [3,4]. Representative bioactive phytochemicals belonging to these classes, including polyphenols and terpenoids, have demonstrated broad pharmacological and nutraceutical potential in experimental models of chronic diseases [5].

Among these, polyphenols constitute one of the most extensively investigated groups because of their antioxidant and anti-inflammatory properties, which are primarily associated with the redox activity of their phenolic structure [6]. Several members of this family, including quercetin and resveratrol derivatives, have shown significant therapeutic potential against oxidative stress-, inflammation-, and cancer-related disorders, further highlighting the relevance of polyphenols as nutraceutical compounds [7,8]. Accordingly, polyphenol-rich nutraceuticals have emerged as promising dietary strategies for the prevention and management of metabolic disorders, including obesity and type 2 diabetes mellitus (T2DM), conditions characterized by oxidative stress, chronic low-grade inflammation, and gut microbiota dysbiosis. However, the application of many polyphenols in functional foods is often limited by their poor water solubility and thermal instability. Recent advances in food technology have addressed these challenges through the development of fortified formulations, including yogurt enriched with protein nanoparticles, which improve polyphenol stability, bioavailability, and antioxidant capacity while expanding their use in functional nutrition [9].

Among naturally occurring polyphenols, phlorizin is a dihydrochalcone glucoside derived from phloretin and is predominantly found in the genus *Malus*, particularly in the peel, roots, and bark of apple trees [10,11]. Like other dihydrochalcones, phlorizin is characterized by an open-chain C-ring structure [12]. Its glycosylated hydroxyl groups enhance water solubility, chemical stability, and transport within both plant and mammalian tissues compared with the corresponding aglycone, phloretin [10,11]. In plants, phlorizin plays a dual biological role. Under physiological conditions, it functions as a constitutive phenolic compound involved in defense against bacterial, fungal, and viral pathogens [12]. Conversely, excessive accumulation of phlorizin in the rhizosphere contributes to Apple Replant Disease (ARD), acting as a major allelochemical responsible for replant-associated growth inhibition. Elevated phlorizin levels induce oxidative stress, promote reactive oxygen species (ROS) accumulation, lipid peroxidation, and membrane damage, as reflected by increased malondialdehyde (MDA) content and relative electrolyte leakage. These alterations impair chlorophyll biosynthesis, photosynthetic performance, and mineral nutrient uptake, ultimately suppressing plant growth and development [13].

Despite the growing body of evidence on the biological activities of phlorizin, important gaps remain in the current scientific literature. Previous studies have largely focused on specific aspects of phlorizin, such as its occurrence in apple tissues, antioxidant and metabolic effects, or its potential role in the management of metabolic disorders, while the available evidence remains fragmented across plant biology, food science, pharmacology, and nutritional research. In particular, a comprehensive integration of the botanical and horticultural aspects of phlorizin with its chemical characteristics, biosynthesis, distribution within apple tissues, physiological and ecological functions, and potential nutraceutical and pharmacological applications is still lacking. Moreover, although several reviews have addressed the health-promoting properties of phlorizin and related apple polyphenols, the evolution of the evidence from early chemical and botanical studies to more recent mechanistic and translational research has not been systematically considered.

Therefore, the aim of the present review is to provide an integrated and updated analysis of phlorizin, encompassing its chemical structure and properties, biosynthetic pathways, occurrence and distribution in different apple tissues, physiological and defensive functions in plants, role in Apple Replant Disease, and reported biological activities and potential applications in human health and functional foods. Particular attention is given to the progression of knowledge from foundational studies to recent experimental evidence, with emphasis on the mechanisms underlying the biological effects of phlorizin and on the relationship between its plant-derived functions and its potential nutraceutical relevance. In contrast to previous reviews, the present work adopts a multidisciplinary perspective that explicitly connects plant biology and horticultural science with food science, pharmacology, and nutritional research. By bringing together these complementary fields, this review aims to identify areas in which evidence is well established, highlight unresolved questions and methodological limitations, and provide a framework for future research on phlorizin and its potential applications.

## 2. Material and Methods

### 2.1. Review Approach and Scope

This review was designed as an integrative narrative review aimed at providing a comprehensive and critical overview of phlorizin, from its occurrence and biological role in apple trees to its potential nutraceutical, functional food, and biomedical applications. In contrast to previous reviews that have primarily focused on the biosynthesis, distribution, bioavailability, or pharmacological properties of phlorizin and other dihydrochalcones [14,15,16,17], the present work adopts a multidisciplinary perspective integrating plant biology, horticultural determinants, molecular mechanisms, biological activities, food technology, and advanced delivery systems.

The literature was examined with particular attention to studies addressing: (i) the occurrence, distribution, and accumulation of phlorizin in *Malus* species; (ii) its biosynthesis and the genetic and enzymatic mechanisms involved; (iii) the influence of genotype, tissue, developmental stage, environmental conditions, and cultivation practices on phlorizin content; (iv) the biological activities and molecular mechanisms associated with phlorizin; and (v) its incorporation into dietary formulations, functional foods, nanostructured systems, and biomaterials.

### 2.2. Literature Identification and Study Selection

A structured literature search was performed to identify and critically evaluate scientific evidence concerning phlorizin (also referred to as phloridzin, phlorhizin, or phloridzin glucoside), with particular emphasis on its occurrence and metabolism in *Malus* species, biosynthesis and distribution, physicochemical properties, biological activities, molecular mechanisms, bioavailability, nutraceutical potential, functional-food applications, and emerging delivery systems. Relevant scientific literature was identified through a structured examination of publications retrieved from PubMed, Scopus, Web of Science, and Google Scholar, using combinations of the terms *phlorizin*, *phloridzin*, *phlorhizin*, and *phloridzin glucoside* with terms related to the main areas investigated, including apple, Malus, biosynthesis, distribution, bioavailability, antioxidant, anti-inflammatory, antidiabetic, SGLT, metabolism, microbiota, neuroprotection, cardioprotection, hepatoprotection, anticancer, wound healing, functional food, nanoparticles, nanofibers, hydrogels, encapsulation, and delivery systems. Publications identified through the reference lists of relevant articles were also considered when they provided additional primary evidence or important historical, chemical, botanical, or mechanistic information.

The literature considered spanned 1996 to 2026, encompassing foundational studies on polyphenol chemistry and phlorizin biology as well as recent experimental investigations published up to 2026. Within this broader period, particular attention was given to the literature specifically focused on phlorizin/phloridzin published from 2005 to 2026, corresponding to the period beginning with the review by Ehrenkranz et al. [14], which represents an important reference point in the consolidation of knowledge specifically concerning this compound. Studies published before 2005 were nevertheless retained when they provided relevant historical, chemical, botanical, biochemical, or methodological evidence necessary to understand the occurrence, characterization, biosynthesis, metabolism, or biological properties of phlorizin. Thus, the temporal range of 1996–2026 represents the overall body of literature considered, rather than a restriction to studies specifically focused on phlorizin throughout the entire period.

Eligible literature included original experimental studies conducted in vitro, ex vivo, in vivo, and in silico, as well as studies addressing food formulation, food technology, encapsulation, nanoparticles, nanofibers, hydrogels, and other advanced delivery systems in which phlorizin was the investigated bioactive compound. Particular emphasis was placed on primary experimental studies providing biological or mechanistic evidence. Studies were considered across different experimental levels, including cell-free and biochemical assays, computational approaches, in vitro cellular models, ex vivo tissues, animal models, and human studies when available. Particular attention was given to studies in which phlorizin itself was the experimental compound; studies exclusively investigating phloretin or unrelated apple polyphenol extracts were not considered direct evidence for the biological effects of phlorizin.

Previous narrative and comprehensive reviews were used primarily to establish the historical and conceptual framework, identify major areas of investigation, consolidate mechanistic information, and locate additional primary studies. In contrast, interpretation of biological effects, molecular mechanisms, and translational potential was based preferentially on the corresponding primary studies. The literature was subsequently organized according to the biological activity investigated, experimental model, molecular mechanism, and potential application reported in each study.

This methodological approach was designed to distinguish the present review from previous syntheses of the field. In particular, a 2021 review had already addressed major aspects of phlorizin, including its biosynthesis, distribution, extraction, bioavailability, and pharmacological activities, whereas a 2024 review provided a more recent general synthesis of its biological activities and underlying mechanisms. Accordingly, the distinctive contribution of the present review is not limited to a bibliographic update but lies in the critical integration of the horticultural and plant-biology perspective with biochemical, nutraceutical, biomedical, functional-food, and advanced-delivery perspectives, together with the selective assessment of studies in which phlorizin represents the actual investigated bioactive compound. This integrative framework enables the evidence to be considered across the continuum from plant occurrence, biosynthesis, and chemical characteristics to biological activity, molecular mechanisms, nutritional and biomedical relevance, technological applications, and emerging strategies for improving its bioavailability and delivery.

### 2.3. Evidence Characterization

To avoid conflating experimental observations with clinically established effects, the evidence was considered according to the experimental level at which each biological activity had been demonstrated. Evidence derived from biochemical or computational analyses was distinguished from findings obtained in cellular, ex vivo, and animal models. Human evidence was considered separately whenever available.

For each biological activity, particular attention was given to the molecular targets and signaling pathways proposed to mediate the observed effects. These included, among others, SGLT1/SGLT2 inhibition, Keap1–Nrf2 signaling, TLR4/MyD88/NF-κB and MAPK pathways, AMPK/PPARα/CPT1a-dependent metabolic regulation, DAF-16/FOXO and SKN-1 stress-response pathways, and microbiota-associated mechanisms. The reported outcomes were interpreted in relation to the experimental model and were not considered evidence of clinical efficacy unless supported by human studies.

### 2.4. Data Synthesis

The extracted information was qualitatively synthesized according to four major domains: plant occurrence and regulation of phlorizin; biological activities and molecular mechanisms; nutraceutical and functional food applications; and advanced delivery and biomaterial-based approaches.

A structured summary of the principal biological activities, experimental models, molecular mechanisms, and biological outcomes was used to facilitate comparison across studies. Particular attention was paid to the consistency of findings across experimental systems, the level of biological validation, and the limitations affecting translation to human applications.

Because of the substantial heterogeneity of experimental models, doses, formulations, biological endpoints, and study designs, a quantitative meta-analysis was not considered appropriate. The review therefore focuses on qualitative integration and critical interpretation of the available evidence, with explicit distinction between preclinical findings and evidence obtained in humans.

## 3. Chemical Structure, Physicochemical Properties and Biosynthesis of Phlorizin

### 3.1. Chemical Structure of Phlorizin

**Figure 1:**
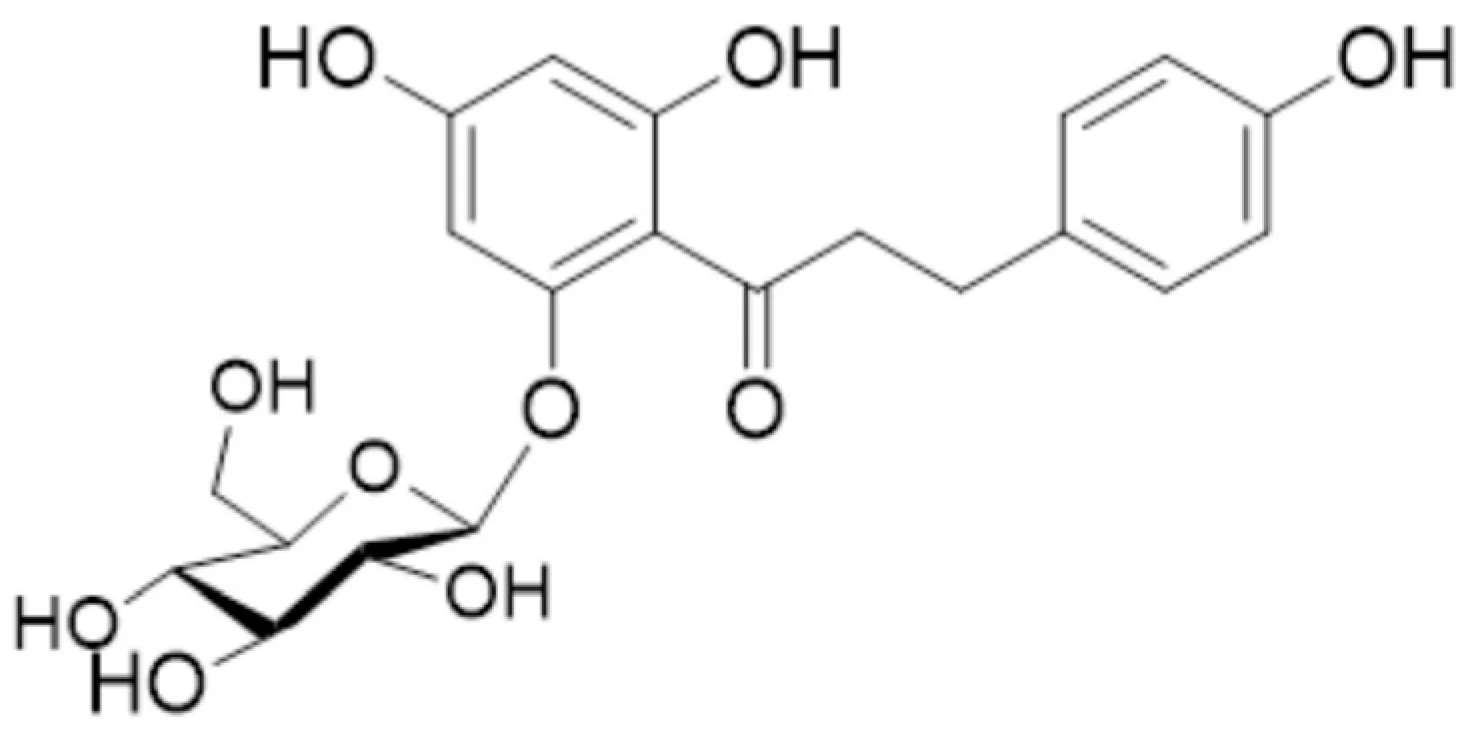
Chemical structure of phlorizin.

Phlorizin (also known as phloridzin), is the major naturally occurring dihydrochalcone glycoside found in apple (*Malus × domestica* Borkh.) and closely related *Malus* species. Since its first isolation from apple root bark in 1835, it has attracted considerable attention because of its importance in both plant physiology and human health. Owing to its highly restricted occurrence within the tribe *Maleae* (*Rosaceae*), phlorizin is regarded as one of the most characteristic phenolic compounds of apple and a distinctive marker of the specialized phenylpropanoid metabolism of the genus *Malus* [14,15,16]. Chemically, phlorizin, (1-[2,4-dihydroxy-6-[(2S,3R,4R,5S,6R)-3,4,5-trihydroxy-6-(hydroxymethyl)tetrahydropyran-2-yl]oxyphenyl]-3-(4-hydroxyphenyl)propan-1-one (Figure 1), belongs to the dihydrochalcone subclass of flavonoids and is formally identified as phloretin 2′-O-β-D-glucopyranoside. It consists of the aglycone phloretin linked to a β-D-glucose molecule through a β-O-glycosidic bond at the 2′ hydroxyl group of ring A. Its molecular formula is C_21_H_24_O_10_ (436.41 g mol^−1^), whereas the aglycone phloretin has the formula C_15_H_14_O_5_ (274.27 g mol^−1^) [17,18].

Unlike classical flavonoids, phlorizin possesses a linear C6–C3–C6 skeleton composed of two aromatic rings connected by a saturated propanone bridge, resulting from the reduction in the α,β-unsaturated double bond characteristic of chalcones. This saturated linker increases molecular flexibility and modifies physicochemical properties such as membrane permeability, protein binding and biological activity compared with other flavonoids [18,19,20].

The molecule contains four phenolic hydroxyl groups, which contribute to hydrogen bonding, metal chelation and antioxidant activity. However, because ring B contains only one hydroxyl substituent and lacks the *ortho*-dihydroxyl arrangement found in compounds such as quercetin or chlorogenic acid, phlorizin generally exhibits lower intrinsic antioxidant capacity than other major apple phenolics [17,21]. The glucose residue markedly increases water solubility, molecular stability and intracellular transport while reducing hydrophobicity and protecting the aglycone from oxidative degradation and potential cytotoxicity [17,18,22].

The structural role of glycosylation is also fundamental for biological activity. The glucose moiety is essential for the recognition of mammalian SGLT1 and SGLT2, whereas the aromatic backbone contributes to transporter affinity. Removal of the glucose residue drastically reduces inhibitory activity, highlighting the importance of glycosylation for the biological function of phlorizin [14,18,23].

Within plant cells, phlorizin is predominantly stored in vacuoles following transporter-mediated sequestration, whereas its aglycone, phloretin, is synthesized transiently in the cytoplasm before rapid glucosylation. This compartmentalization facilitates long-term storage, minimizes oxidative degradation and allows rapid mobilization under stress conditions, although direct evidence of intracellular trafficking remains limited [15,17,24].

From an evolutionary perspective, phlorizin has a remarkably restricted distribution compared with other flavonoids, accumulating mainly in *Malus* species. This taxonomic specificity suggests that the biosynthetic pathway responsible for dihydrochalcone formation evolved relatively late through the recruitment of additional enzymes within the phenylpropanoid pathway [15,24]. Metabolomic analyses further indicate that phlorizin is the predominant member of a larger family of apple dihydrochalcones, including the aglycone phloretin and the glucosylated derivatives trilobatin and sieboldin, both of which contain a glucose moiety, as well as several other glycosylated derivatives. The abundance of these compounds varies among tissues, cultivars, and wild *Malus* species, reflecting differences in glycosyltransferase activity and pathway regulation [17,25].

Because of its unique distribution, phlorizin is widely recognized as an important chemotaxonomic marker that distinguishes *Malus* species from other members of the Rosaceae family. Its characteristic accumulation reflects the specialized evolution of apple secondary metabolism and supports its application in germplasm characterization and breeding programs aimed at improving disease resistance and phytochemical quality [15,24,26].

### 3.2. Physicochemical Properties of Phlorizin

The physicochemical properties of phlorizin largely determine its biological functions in planta, its technological behaviour during food processing, and its potential applications in nutraceutical and pharmaceutical formulations. These properties are primarily governed by the molecular architecture of the compound, particularly the presence of multiple hydroxyl groups, the β-D-glucosyl residue, and the saturated dihydrochalcone backbone. Together, these structural features influence polarity, solubility, stability, redox behavior, molecular interactions and bioavailability [15,17,18].

Unlike many flavonoid aglycones, phlorizin exhibits relatively high polarity due to the presence of the glucose moiety. Glycosylation substantially increases its hydrophilicity compared with phloretin, facilitating intracellular transport and vacuolar accumulation in plant tissues. This increased polarity also explains why phlorizin is readily extracted using aqueous alcohols, particularly methanol–water and ethanol–water mixtures, which remain the solvents of choice for analytical determination of apple phenolics [27,28,29,30].

Nevertheless, despite being classified as a glycosylated phenolic compound, phlorizin cannot be considered highly water soluble. The extensive aromatic backbone still confers a certain degree of hydrophobicity, resulting in an amphiphilic molecular behaviour. Consequently, extraction efficiency depends strongly on solvent composition, temperature and extraction technique. Hydroalcoholic mixtures containing 60–80% methanol or ethanol generally provide the highest recoveries, whereas extraction with pure water is considerably less efficient [18,28,29].

This amphiphilic nature has important biological implications. In plant cells, phlorizin can diffuse within aqueous compartments while maintaining sufficient affinity for biological membranes to participate in interactions with membrane-associated proteins. In mammalian systems, however, the glucose residue reduces passive diffusion across intestinal epithelial membranes, making enzymatic hydrolysis by intestinal β-glucosidases a prerequisite for efficient absorption. Following hydrolysis, the resulting aglycone phloretin displays substantially greater membrane permeability because of its increased lipophilicity [14,17,18].

#### 3.2.1. Stability as Affected by pH

The chemical stability of phlorizin is strongly influenced by environmental pH. Under mildly acidic conditions, similar to those naturally occurring in apple tissues (pH 3.0–4.5), the molecule remains relatively stable, allowing long-term accumulation during fruit development and storage [15,29].

As pH increases, however, deprotonation of phenolic hydroxyl groups enhances molecular reactivity, promoting oxidation and subsequent degradation. Alkaline conditions facilitate the formation of phenoxy radicals and quinone intermediates, which may undergo polymerization or react with proteins and other phenolic compounds. Consequently, degradation rates increase markedly under alkaline environments, explaining why alkaline extraction procedures are generally avoided for quantitative determination of phlorizin [18,21].

The pH-dependent stability of phlorizin also has practical implications for food processing. Apple juices, ciders and fermented products generally maintain an acidic pH that contributes to preserving dihydrochalcone integrity. Conversely, processing conditions involving alkaline treatments may substantially reduce phlorizin concentrations through oxidative degradation.

#### 3.2.2. Thermal Stability

Compared with anthocyanins, phlorizin exhibits relatively good thermal stability. The absence of the highly reactive flavylium cation characteristic of anthocyanins makes the molecule less susceptible to heat-induced structural rearrangements. Nevertheless, prolonged thermal treatments progressively decrease phlorizin concentration through oxidation, hydrolysis and secondary degradation reactions [17,18].

Several studies investigating apple processing have demonstrated that moderate thermal treatments used during pasteurization generally preserve a large proportion of native phlorizin, whereas extended heating or repeated thermal exposure significantly reduce its concentration. The degradation rate is further accelerated in the presence of oxygen or transition metal ions, which catalyze oxidation reactions through free radical formation [18,21].

Interestingly, the thermal behavior of phlorizin differs from that of phloretin. While glycosylation improves water solubility, it also contributes to increased thermal stability by reducing molecular reactivity. This protective effect represents another example of the multifunctional role played by glycosylation in plant secondary metabolism.

#### 3.2.3. Light and Oxygen Sensitivity

Like most phenolic compounds, phlorizin is susceptible to photooxidation. Exposure to ultraviolet radiation or prolonged visible light promotes the generation of ROS capable of oxidizing phenolic hydroxyl groups. Consequently, storage under dark conditions is generally recommended for analytical standards and extracts intended for quantitative analyses [28,29].

The presence of dissolved oxygen further accelerates degradation through auto-oxidative mechanisms. Oxidative reactions may be catalyzed enzymatically by polyphenol oxidases (PPOs) naturally present in damaged apple tissues. Although phlorizin is generally considered a poorer PPO substrate than catechol-containing phenolics such as chlorogenic acid, enzymatic oxidation still contributes to postharvest changes in phenolic composition following tissue disruption.

This phenomenon has important implications for sample preparation. Rapid freezing, extraction under reduced oxygen availability and addition of antioxidant stabilizers are commonly employed to minimize oxidative losses during metabolomic analyses.

#### 3.2.4. Redox Properties and Antioxidant Behaviour

The antioxidant activity of phlorizin originates primarily from hydrogen atom donation by phenolic hydroxyl groups and subsequent stabilization of phenoxy radicals through resonance. However, the molecular structure of phlorizin lacks the *ortho*-dihydroxyl configuration generally regarded as the most effective structural determinant of radical scavenging activity [16,21,31].

Comparative studies consistently rank phlorizin below quercetin, catechin, epicatechin and chlorogenic acid in terms of radical scavenging capacity [17]. Nevertheless, its antioxidant properties remain biologically relevant because phlorizin often accumulates at much higher concentrations than other individual phenolics in several apple tissues, particularly bark, leaves and roots [17,20,25,29]. Therefore, its quantitative abundance partially compensates for its lower intrinsic antioxidant potency.

Beyond direct radical scavenging, phlorizin also contributes indirectly to cellular redox homeostasis by modulating antioxidant enzymes and signaling pathways. Experimental studies have reported effects on superoxide dismutase (SOD), catalase (CAT) and glutathione peroxidase activities, although most available evidence derives from mammalian systems rather than plant physiology [17,32].

#### 3.2.5. Interactions with Proteins and Biological Membranes

One of the most remarkable physicochemical characteristics of phlorizin is its capacity to interact with membrane proteins. The molecule combines a hydrophilic glucose residue with a relatively hydrophobic aromatic backbone, allowing simultaneous interactions with polar and nonpolar regions of biological macromolecules.

This dual affinity explains the exceptionally high inhibitory activity of phlorizin against SGLT1 and SGLT2. Structural studies indicate that the glucose moiety is primarily responsible for transporter recognition, whereas hydrophobic interactions involving the aromatic rings stabilize ligand binding [14,18].

Within plant tissues, comparable interactions may occur with membrane transporters involved in vacuolar sequestration of phenolic metabolites, although these mechanisms remain poorly understood.

#### 3.2.6. Bioavailability, Metabolism, Pharmacokinetics, and Safety

Following oral administration, phlorizin can undergo hydrolysis of its β-glycosidic bond in the gastrointestinal tract, generating phloretin and glucose. Phloretin is subsequently absorbed and extensively subjected to phase II metabolism, particularly glucuronidation and sulfation. Consequently, systemic exposure to intact phlorizin is expected to be limited, whereas phloretin and its conjugated metabolites may represent the predominant circulating forms. This conversion complicates the attribution of biological effects exclusively to the parent compound. In addition, the doses and routes of administration used in experimental models vary considerably, and parenteral treatments bypass gastrointestinal metabolism and cannot be directly compared with dietary exposure. Available long-term human safety data are insufficient. Because phlorizin inhibits both intestinal SGLT1 and renal SGLT2, concentrated or chronic exposure may affect intestinal glucose absorption, renal glucose handling, and fluid balance. Standardized pharmacokinetic, dose–response, interaction, and long-term safety studies are therefore required before nutraceutical efficacy or safety can be established [15,17,30].

### 3.3. Biosynthetic Pathway of Phlorizin

Phlorizin biosynthesis is a highly specialized branch of the phenylpropanoid pathway that has evolved almost exclusively in the genus *Malus*. Unlike the major flavonoid subclasses, its restricted distribution results from specialized enzymatic activities that redirect carbon flux from the general phenylpropanoid pathway toward dihydrochalcone production [15]. Significant advances in enzymology and functional genomics have clarified the pathway, identifying the key enzymatic steps and the genes encoding the reductases and glycosyltransferases involved [15,17,20,33,34].

**Figure 2:**
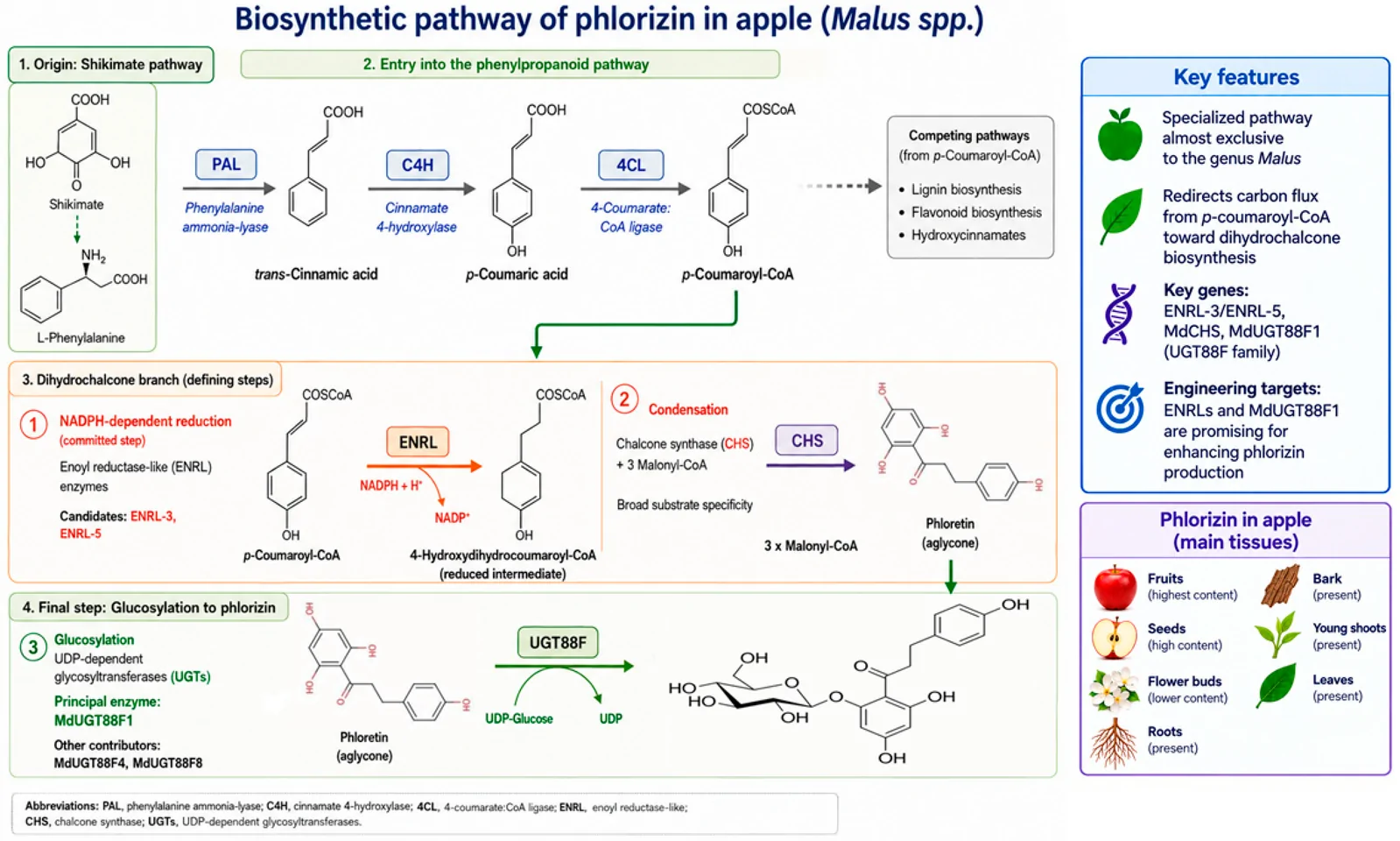
Biosynthetic pathway of phlorizin.

Phlorizin biosynthesis originates from the shikimate pathway, where L-phenylalanine is converted into *trans*-cinnamic acid by phenylalanine ammonia-lyase (PAL), the major regulatory enzyme controlling carbon allocation into phenylpropanoid metabolism. Subsequent reactions catalysed by cinnamate 4-hydroxylase (C4H) and 4-coumarate:CoA ligase (4CL) generate *p*-coumaroyl-CoA, the common precursor of lignin, flavonoids and dihydrochalcones. In apple, a portion of *p*-coumaroyl-CoA is diverted toward dihydrochalcone biosynthesis instead of conventional flavonoid formation [15,17,24] (Figure 2).

The defining step of this pathway is the NADPH-dependent reduction of *p*-coumaroyl-CoA to 4-hydroxydihydrocoumaroyl-CoA, representing the first committed reaction toward dihydrochalcone biosynthesis. Members of the enoyl reductase-like (ENRL) family, particularly ENRL-3 and ENRL-5, have been proposed as candidate enzymes, although definitive biochemical evidence is still lacking [15,17,35].

The reduced intermediate is then condensed with three malonyl-CoA molecules by chalcone synthase (CHS) to produce phloretin, the aglycone precursor of phlorizin. CHS, the same enzyme involved in flavonoid biosynthesis, exhibits broad substrate specificity, indicating that metabolic innovation in apple resulted from substrate diversification rather than the evolution of a novel enzyme. High expression of *MdCHS* correlates with elevated phlorizin accumulation, whereas reduced CHS activity decreases both flavonoid and dihydrochalcone biosynthesis [15,24,33] (Figure 2).

The final and decisive step is the glucosylation of phloretin to phlorizin by UDP-dependent glycosyltransferases (UGTs). MdUGT88F1 has been identified as the principal phloretin-specific glycosyltransferase, exhibiting high substrate specificity compared with other flavonoids [33,34,36]. Additional UGT88F family members, including MdUGT88F4 and MdUGT88F8, also contribute to phloretin glucosylation but differ in catalytic efficiency and tissue-specific expression [36]. Structural engineering of MdUGT88F1 has further demonstrated that this enzyme represents a major target for enhancing biotechnological phlorizin production [37] (Figure 2).

## 4. Distribution of Phlorizin in Malus Species and Related Horticultural Taxa

### 4.1. Distribution of Phlorizin Among Malus Species

The restricted phylogenetic distribution of phlorizin represents one of the most distinctive features of this dihydrochalcone and has attracted considerable attention from plant biochemists, taxonomists and horticultural scientists. Unlike flavonols, anthocyanins or hydroxycinnamic acids, which are ubiquitous throughout higher plants, phlorizin accumulates predominantly in species belonging to the genus *Malus* and, to a lesser extent, in a few closely related members of the tribe *Maleae* (*Rosaceae*). This peculiar distribution strongly suggests that the capacity to synthesize dihydrochalcones arose through lineage-specific evolution of the phenylpropanoid pathway after the diversification of the *Rosaceae* family [15,17].

Comprehensive phytochemical investigations have consistently demonstrated that apple trees represent by far the richest natural source of phlorizin identified to date. Although the compound can occasionally be detected in trace amounts in other *Rosaceae* species, its concentration is generally several orders of magnitude lower than that found in *Malus*. Consequently, phlorizin has been proposed as a reliable chemotaxonomic marker for the genus, complementing molecular markers used in phylogenetic and breeding studies [15,17,38].

#### 4.1.1. Phlorizin in Cultivated Apple (*Malus × domestica*)

Among cultivated fruit crops, *Malus × domestica* exhibits the highest and most consistent accumulation of phlorizin. The metabolite is present throughout the plant, including roots, bark, young shoots, leaves, flower buds, fruits and seeds, although its concentration varies considerably among organs and developmental stages. Early phytochemical studies already recognized apple bark as one of the richest botanical sources of phlorizin, a finding later confirmed by chromatographic and metabolomic analyses using HPLC-DAD and LC-MS/MS [15,17,27].

The widespread distribution of phlorizin throughout the apple tree suggests that the compound fulfills multiple physiological functions rather than serving merely as a storage metabolite. High constitutive concentrations in perennial organs such as roots and bark support the hypothesis that phlorizin contributes to constitutive defense mechanisms against soil-borne pathogens and herbivores. Conversely, its accumulation in young expanding tissues indicates an additional role in protecting metabolically active organs from oxidative stress and environmental challenges.

Recent metabolomic studies have further demonstrated that phlorizin is one of the dominant soluble phenolics in vegetative tissues of cultivated apple, frequently accounting for a substantial proportion of the total dihydrochalcone pool. Nevertheless, its relative abundance within the overall phenolic profile differs markedly according to genotype, tissue and environmental conditions, reflecting the complex regulation of its biosynthetic pathway [17,38].

#### 4.1.2. Distribution Among Wild *Malus* Species

Wild apple species constitute an invaluable reservoir of genetic diversity for both breeding and evolutionary studies. Comparative metabolomic analyses have revealed that the capacity to synthesize phlorizin is conserved across numerous wild *Malus taxa*, including *M. sieversii*, *M. baccata*, *M. orientalis*, *M. prunifolia* and *M. micromalus*, although substantial quantitative differences exist among species [17,25,39].

Particularly noteworthy is *Malus sieversii*, the primary progenitor of the cultivated apple. Native to the forests of Central Asia, this species exhibits remarkable variation in phlorizin concentration among natural populations [25]. Such variability reflects the extraordinary genetic diversity maintained within wild germplasm and provides valuable opportunities for identifying alleles controlling dihydrochalcone biosynthesis. Indeed, metabolomic and genomic analyses increasingly exploit *M. sieversii* populations to investigate the evolutionary origin of specialized phenolic metabolism and to identify candidate genes for marker-assisted breeding [17,25].

Similarly, *M. baccata*, which is widely used as a rootstock in northern regions because of its exceptional cold tolerance, often exhibits elevated phlorizin concentrations in roots and bark [17]. This observation has led several authors to speculate that constitutively high dihydrochalcone levels may contribute to enhanced resistance against abiotic stress and root-associated pathogens, although direct experimental evidence remains limited.

#### 4.1.3. Chemotaxonomic, Evolutionary and Breeding Significance of Phlorizin in *Malus*

Phlorizin is a highly restricted secondary metabolite whose distribution is largely confined to the genus *Malus*. This marked taxonomic specificity gives the compound considerable chemotaxonomic value, as its biosynthesis reflects the evolution of a specialized metabolic branch within the phenylpropanoid network. Comparative metabolomic analyses consistently identify phlorizin as one of the principal metabolites responsible for the biochemical discrimination of *Malus* from closely related genera such as *Pyrus*, *Cydonia*, and *Chaenomeles*, often accounting for a substantial proportion of the separation observed in multivariate analyses [15,17,29,40]. Genomic evidence further supports this specialization, as enzymes involved in phlorizin biosynthesis, particularly members of the UGT88F family, show lineage-specific expansion within *Malus*, suggesting adaptive evolution related to pathogen defence, oxidative stress, and environmental adaptation [34,36]. Although all investigated *Malus* species produce phlorizin, its concentration varies considerably among cultivated and wild genotypes. These differences are associated with variation in the expression of phenylpropanoid biosynthetic genes (PAL, C4H, 4CL and CHS), the activity of phloretin glycosyltransferases such as MdUGT88F1, precursor availability, and regulatory mechanisms acting beyond transcriptional control [24,34]. The conservation of the biosynthetic pathway across diverse *Malus* species indicates that phlorizin biosynthesis originated before the diversification of the genus, whereas subsequent quantitative variation likely reflects adaptation to different ecological niches. Species adapted to colder environments or exposed to higher pathogen pressure frequently accumulate greater amounts of dihydrochalcones, supporting a role for phlorizin in environmental resilience [15,17]. This natural variability has important implications for apple breeding and germplasm conservation. Wild *Malus* germplasm represents an important source of genetic diversity for identifying elite genotypes with enhanced phlorizin biosynthesis [25,39]. The integration of metabolomic phenotyping with genome-wide association studies (GWAS), genomic selection and functional characterization of key biosynthetic genes may facilitate the development of cultivars combining high fruit quality, improved phytochemical composition, and enhanced tolerance to abiotic and biotic stresses [17,33,34].

## 5. Genetic and Horticultural Factors Affecting Phlorizin Accumulation

### 5.1. Cultivar and Genotype

Genotype is widely recognized as the principal determinant of phlorizin accumulation in apple. Although environmental conditions and orchard management significantly modulate the concentration of this dihydrochalcone, numerous studies have demonstrated that the genetic background exerts the strongest influence on both constitutive and inducible phlorizin biosynthesis [17,25,33,39]. Consequently, substantial quantitative differences can be observed not only among *Malus* species but also among cultivars of *Malus × domestica*, breeding selections, rootstocks and wild accessions. The predominance of genotype is not unexpected considering the highly specialized nature of the phlorizin biosynthetic pathway. Unlike chlorogenic acid, catechins or quercetin glycosides, whose biosynthetic enzymes are highly conserved throughout angiosperms, phlorizin biosynthesis depends on lineage-specific modifications of the phenylpropanoid pathway and on specialized UDP-glycosyltransferases that evolved within the genus *Malus* [15,17,41]. Consequently, genetic polymorphisms affecting biosynthetic enzymes, regulatory transcription factors or precursor availability can produce large differences in metabolite accumulation.

#### 5.1.1. Natural Genetic Variability Within the Genus *Malus*

One of the most comprehensive evaluations of natural variation in phlorizin content was conducted using the USDA *Malus* germplasm collection. In this study, 345 accessions representing 29 species and 16 interspecific hybrids were analyzed over three consecutive growing seasons. Leaf phlorizin concentration ranged from 17.3 to 113.7 mg g^−1^ dry weight, demonstrating exceptionally broad genetic variability. Statistical analysis showed that genotype explained approximately 62% of the total phenotypic variation, whereas year-to-year correlations remained high (r ≈ 0.70), indicating that phlorizin accumulation is a relatively stable inherited trait rather than a transient environmental response [25,33,39]. Interestingly, the survey also revealed marked qualitative differences in dihydrochalcone composition. While approximately 80% of the analyzed accessions accumulated phlorizin as the predominant dihydrochalcone, other genotypes accumulated substantial amounts of trilobatin and sieboldin, indicating that different *Malus* lineages have evolved distinct glycosylation patterns [25,39]. These findings suggest that natural selection has acted not only on the quantity but also on the qualitative composition of dihydrochalcones during the evolution of the genus. From a breeding perspective, this diversity represents an invaluable resource because it provides naturally occurring alleles controlling both total phlorizin accumulation and the relative abundance of structurally related metabolites [17,39].

#### 5.1.2. Differences Among Cultivated Cultivars

Significant variation in phlorizin accumulation has been reported among cultivated apple varieties, with cultivars such as ‘Golden Delicious’, ‘Royal Gala’, ‘Fuji’, ‘Granny Smith’, ‘Braeburn’, and ‘Jonathan’ differing in both phlorizin concentration and tissue distribution even under similar environmental conditions, confirming a strong genetic component in the regulation of dihydrochalcone metabolism [27,28]. Recent metabolomic studies have further demonstrated that phlorizin accumulation is highly tissue-specific, reflecting differential regulation of biosynthetic genes among plant organs [17,40]. In addition, phlorizin content does not necessarily correlate with the abundance of other phenolic compounds, likely because different branches of the phenylpropanoid pathway compete for common precursors [15,24]. Comparisons between traditional or heritage cultivars and modern commercial cultivars indicate that some traditional genotypes may accumulate higher levels of phlorizin, possibly reflecting breeding practices that have prioritised fruit yield and quality over secondary metabolism. Nevertheless, considerable variability occurs within both groups, further supporting the strong influence of genotype on phlorizin accumulation [17,25,39,40]. These findings highlight the value of traditional germplasm as a genetic resource for improving phlorizin biosynthesis in future apple breeding programs [38,39].

#### 5.1.3. Molecular Basis of Genotypic Variation

The molecular mechanisms underlying cultivar differences have become considerably clearer during the past decade. Genome-wide analyses identified MdUGT88F1 and its closely related paralogue MdUGT88F4 as the principal enzymes catalysing the glucosylation of phloretin to phlorizin [33,36,41]. Expression analyses across numerous *Malus* species and cultivars demonstrated that transcript abundance of these genes closely correlates with leaf phlorizin concentration, strongly supporting their role as major determinants of natural metabolic variation [33,34]. Functional studies further confirmed this hypothesis. RNA interference-mediated silencing of MdUGT88F1 significantly reduced phlorizin accumulation while increasing phloretin and trilobatin concentrations, demonstrating that the terminal glycosylation step represents a major metabolic bottleneck [24,34,37]. Conversely, overexpression of the same gene enhanced phlorizin biosynthesis and modified carbon partitioning, chloroplast development and disease resistance [34]. Structural engineering studies have further identified UGT88F1 as a promising target for improving phlorizin biosynthesis through metabolic engineering [37].

#### 5.1.4. Genotype × Environment Interactions

Although genotype explains most of the observed variation, phlorizin accumulation should be regarded as a quantitative trait resulting from strong genotype × environment interactions [17,25]. Identical cultivars grown under different climatic conditions frequently exhibit significant differences in phlorizin concentration, whereas different cultivars cultivated within the same orchard generally maintain their relative ranking. Recent transcriptomic evidence indicates that environmental cues—including pathogen attack, nutrient availability and abiotic stress—modify the expression of MdUGT88F1, MdUGT88F4, CHS and upstream phenylpropanoid genes, thereby influencing the phenotypic expression of high-phlorizin genotypes [24,33,34]. These observations have important implications for breeding programmes. Selection based solely on phenotypic measurements obtained in a single environment may underestimate the true genetic potential of individual genotypes. Multi-environment trials integrating metabolomics, genome-wide association studies and genomic prediction will therefore be essential for developing cultivars characterized by stable phlorizin accumulation under contrasting climatic conditions [17,25].

### 5.2. Horticultural Factors

In addition to genetic background and developmental stage, horticultural practices substantially influence phlorizin accumulation in apple trees by modulating carbon allocation, phenylpropanoid metabolism, and plant responses to environmental stress. Since phlorizin is synthesized through the phenylpropanoid pathway and functions as an important antioxidant and defense-related metabolite, orchard management practices that affect tree vigor, photosynthetic activity, nutrient availability, and stress conditions can alter its concentration in different plant organs [15].

#### 5.2.1. Light Exposure and Canopy Management

Light is one of the most important environmental regulators of phenolic metabolism in apple trees. Increased light interception resulting from appropriate pruning, canopy training, or reduced tree density enhances photosynthetic carbon assimilation and stimulates the expression of phenylpropanoid biosynthetic genes. Although most studies have focused on flavonols and anthocyanins, similar regulatory mechanisms are believed to influence dihydrochalcone biosynthesis because phlorizin shares the same upstream metabolic pathway. Consequently, improved canopy illumination generally promotes phlorizin accumulation, particularly in leaves and young shoots where biosynthesis is most active [15,42]. Conversely, prolonged shading reduces photosynthetic capacity and limits carbon availability for secondary metabolism, leading to lower concentrations of phenolic compounds, including phlorizin. The regulation of phlorizin biosynthesis by light is also consistent with its proposed physiological role in protecting photosynthetically active tissues against oxidative stress.

#### 5.2.2. Water, Nutrient, Crop Load, and Harvest Management

Agronomic practices, including water and nutrient management, crop load regulation, pruning, and harvest timing, can influence phlorizin accumulation by modifying plant growth, source–sink relationships, carbon allocation, and secondary metabolism. Moderate environmental or nutritional stress may favor the allocation of carbon toward phenolic secondary metabolites, whereas excessive water availability or nitrogen fertilization may promote vegetative growth and primary metabolism, potentially reducing the relative accumulation of phenolic compounds. Conversely, severe water stress can impair photosynthesis and carbon availability, thereby limiting secondary metabolite biosynthesis. Balanced mineral nutrition, particularly the adequate supply of potassium and phosphorus, may further support photosynthetic activity and carbohydrate transport, although direct evidence linking these factors specifically to phlorizin accumulation remains limited [15].

Similarly, crop load and pruning can modify the balance between reproductive and vegetative sinks and consequently affect the availability of carbon substrates for secondary metabolism. Excessive fruit load may increase sink demand, whereas appropriate fruit thinning and moderate pruning can alter canopy architecture, light interception, and photosynthetic activity, potentially creating more favorable conditions for phlorizin biosynthesis. However, these effects should be interpreted cautiously, as direct experimental evidence specifically addressing phlorizin remains limited [15].

Seasonal development and harvest timing represent additional factors that can substantially affect phlorizin concentration. Phlorizin accumulation varies among plant organs and throughout the growing season, reflecting changes in developmental stage and metabolic activity. In four apple cultivars, bark and leaves generally showed the highest phlorizin concentrations during spring, particularly in May, whereas twigs continued to accumulate phlorizin until late summer or early autumn [40]. These findings highlight the importance of harvest timing when leaves, bark, or pruning residues are considered as potential sources of phlorizin for nutraceutical or pharmaceutical applications. Seasonal variability should also be taken into account when comparing phlorizin concentrations among cultivars, plant organs, or agronomic management systems, emphasizing the need for standardized sampling and harvesting conditions [40].

#### 5.2.3. Biotic and Abiotic Stress

Numerous studies suggest that phlorizin participates in plant defense against both biotic and abiotic stresses. Environmental factors such as pathogen infection, ultraviolet radiation, temperature fluctuations, and oxidative stress can activate phenylpropanoid metabolism, leading to altered phlorizin accumulation. Although phlorizin alone does not fully explain disease resistance, it contributes to the antioxidant defense system and interacts with other phenolic compounds involved in stress adaptation [43]. Recent molecular studies have further demonstrated that regulation of key biosynthetic genes, including the phloretin-specific glycosyltransferase MdPGT1, determines tissue-specific phlorizin accumulation and mediates plant adaptation to environmental conditions [44,45].

## 6. Biological and Nutraceutical Activities of Phlorizin

Phlorizin exhibits a broad spectrum of biological and nutraceutical activities, including antioxidant, antidiabetic, anti-inflammatory, metabolic, antimicrobial, neuroprotective, cardioprotective, hepatoprotective, ocular, bone-protective, and antitumor effects, as well as emerging applications in functional foods and advanced delivery systems. The experimental evidence, together with the principal models, molecular pathways, and biological outcomes, is summarized in Table 1 and Table 2. Overall, the available literature indicates that many of these effects are interconnected and largely reflect the ability of phlorizin to modulate oxidative stress, inflammation, glucose and lipid metabolism, cellular stress responses, and gut microbiota.

### 6.1. Antioxidant Activity

Phlorizin displays antioxidant activity in both cell-free and biological systems. However, available evidence suggests that its effects in vivo are mainly associated with enhancement of endogenous antioxidant defenses rather than direct radical scavenging. In mammalian models, phlorizin activates the Nrf2-dependent antioxidant response, increasing endogenous antioxidant enzymes and reducing oxidative damage. Similar stress-protective effects have been observed in Caenorhabditis elegans, where activation of DAF-16/FOXO and other conserved stress-response pathways was associated with increased resistance to oxidative stress and longevity [13,46,47,48].

These antioxidant effects are also relevant to tissue protection. In retinal cells, phlorizin attenuated oxidative injury and preserved cell viability, while in aging models it reduced oxidative damage and contributed to the preservation of hippocampal integrity [47,49]. Its incorporation into nanofibrous membranes further enhanced the antioxidant properties of the resulting biomaterials [46]. Thus, antioxidant activity represents a common mechanistic component underlying several of the protective effects described for phlorizin.

### 6.2. Antidiabetic Activity and SGLT Inhibition

Phlorizin is historically recognized as a non-selective inhibitor of sodium–glucose cotransporters SGLT1 and SGLT2 and provided the pharmacological basis for the subsequent development of selective SGLT2 inhibitors [14,50,51]. By interfering with renal glucose reabsorption and intestinal glucose absorption, phlorizin promotes glucose excretion and contributes to improved glucose homeostasis. Additional inhibition of α-glucosidase may further contribute to the attenuation of postprandial glucose excursions.

Experimental studies have extended these observations to diabetic complications and metabolic dysfunction. In diabetic retinopathy models, phlorizin reduced hyperglycaemia, retinal inflammation and pathological angiogenesis while preserving blood–retinal barrier integrity [52]. In diet-induced metabolic dysfunction, it improved glucose tolerance and insulin sensitivity and attenuated dyslipidaemia and excessive weight gain [53]. Its ability to inhibit SGLT-mediated glucose transport has also been demonstrated in extra-renal experimental systems [54].

Despite these pharmacological effects, the poor oral bioavailability, rapid hydrolysis to phloretin, and lack of selectivity of native phlorizin limit its direct clinical application [51]. Its principal relevance therefore remains both as a mechanistic probe of glucose transport and as a structural precursor for the development of SGLT-targeting compounds.

### 6.3. Anti-Inflammatory Activity

Anti-inflammatory activity is another recurrent feature of phlorizin action. Across different experimental models, phlorizin suppresses pro-inflammatory signaling involving TLR4/MyD88/NF-κB and MAPK pathways, resulting in reduced production of major inflammatory mediators [46,47,49,55]. This activity has been observed in neural, ocular, metabolic, and wound-healing contexts, indicating that the anti-inflammatory effects of phlorizin are not restricted to a single tissue.

The interaction between antioxidant and anti-inflammatory mechanisms appears particularly relevant. In wound-healing biomaterials, for example, phlorizin simultaneously enhanced antioxidant defenses and attenuated inflammatory signaling, contributing to tissue repair [55]. Similarly, its effects in aging and ocular models combine reduction in oxidative stress with suppression of inflammatory responses [47,49]. These findings support the concept that modulation of redox-inflammatory balance represents one of the central mechanisms underlying the pleiotropic effects of phlorizin.

### 6.4. Metabolic and Lipid-Regulating Effects

Beyond its established effects on glucose transport, phlorizin acts as a broader metabolic regulator. In high-fat and high-fat/high-fructose diet models, supplementation improved lipid and glucose homeostasis, reduced hepatic steatosis and attenuated excessive body-weight gain. These effects were associated with activation of pathways promoting fatty-acid oxidation and suppression of lipogenic signaling, including AMPKα, PPARα/CPT1a and PPARγ/SREBP-1c/FASN/ACC1 [13,53].

An important component of this metabolic activity involves the intestinal microbiota. Phlorizin partially restores diet-associated microbial dysbiosis and increases the production of short-chain fatty acids, suggesting an interaction between phlorizin metabolism, intestinal microbial ecology, and host metabolic responses [13,53]. The reduction in phlorizin-associated effects following microbiota depletion further supports a functional role for the gut microbiota in its metabolism and biological activity [13,53]. These observations provide a mechanistic link between the metabolic and microbiota-modulating properties of phlorizin.

### 6.5. Antibacterial and Antiviral Activities

Phlorizin has also demonstrated antimicrobial properties, although the evidence remains predominantly experimental. Antibacterial activity has been reported against several Gram-positive and Gram-negative bacteria, including *Staphylococcus aureus*, *Escherichia coli*, *Listeria monocytogenes*, and *Bacillus subtilis* [32,46,56,57,58,59,60]. Proposed mechanisms include interference with bacterial growth, energy metabolism, protein synthesis, and biofilm formation, although these mechanisms require further characterization.

The antimicrobial properties of phlorizin have particular relevance to wound-healing biomaterials, where they may complement its antioxidant and anti-inflammatory activities [46,55]. Computational and ex vivo studies have also suggested antidiarrheal and spasmolytic effects associated with intestinal signaling and smooth-muscle modulation [60], although these findings require in vivo validation.

Limited evidence additionally suggests antiviral activity against experimental BVDV and Zika virus infection. In these models, phlorizin affected host cellular pathways involved in viral replication, including autophagy and glucose metabolism [56,61]. However, the antiviral evidence is currently restricted to a small number of experimental systems and does not yet establish a broader antiviral potential.

### 6.6. Antitumor Activity

The antitumor potential of phlorizin has been investigated mainly in experimental cancer models. Studies have reported inhibition of cancer-cell proliferation, migration, invasion, and survival, with involvement of JAK2/STAT3, mitochondrial apoptotic pathways, and other signaling networks associated with tumor progression [62,63,64,65,66]. Experimental evidence has also been obtained in models of hepatocellular carcinoma, where phlorizin reduced tumor development and improved selected biochemical parameters [64].

These findings suggest that phlorizin may influence tumor biology through a combination of metabolic, inflammatory, and apoptosis-related mechanisms. Nevertheless, current evidence remains predominantly preclinical, and the therapeutic relevance of these observations requires confirmation in more extensive experimental and translational studies.

### 6.7. Hepatoprotective and Further Pleiotropic Protective Effects

The hepatoprotective effects of phlorizin are closely linked to its antioxidant, anti-inflammatory, and metabolic activities. In experimental models of obesity-associated diabetes and NAFLD/NASH, phlorizin improved glucose and hepatic lipid metabolism and reduced steatosis and pathological liver alterations [67]. Additional evidence from an experimental model of acetaminophen-induced liver injury suggests that phlorizin may also protect against acute hepatic damage through activation of antioxidant responses [68].

Other experimental studies have described protective effects in tissues including skin, brain, heart, and intestine [32]. Although these observations reinforce the pleiotropic nature of phlorizin, they should be interpreted primarily as preclinical evidence rather than as evidence of established therapeutic efficacy.

### 6.8. Cardioprotective Effects

Recent experimental evidence indicates that phlorizin may also protect the myocardium under conditions of diet-induced hyperlipidaemia. In this context, its administration improved lipid metabolism and attenuated myocardial oxidative stress, inflammation, apoptosis, and pyroptosis [69]. The involvement of the HK1/NLRP3/Caspase-1 axis provides a potential mechanistic connection between glucose metabolism, oxidative stress, and inflammatory cell death.

These findings extend the biological profile of phlorizin beyond its classical metabolic effects and suggest that modulation of metabolic and redox-inflammatory pathways may contribute to cardiovascular protection. However, the evidence remains limited to experimental models and requires confirmation in independent studies [69].

### 6.9. Neuroprotection and the Microbiota-Gut–Brain Axis

Neuroprotective activity represents one of the most complex emerging properties of phlorizin. Experimental studies indicate that phlorizin can attenuate cognitive and behavioral alterations associated with aging, metabolic dysfunction, and chronic stress through a combination of antioxidant, anti-inflammatory, anti-apoptotic, and synaptic effects [47,53,70].

Importantly, several studies implicate the microbiota–gut–brain axis in these responses. Phlorizin-induced changes in intestinal microbial composition and microbial metabolites have been associated with improved hippocampal function, synaptic integrity, and neuroinflammatory status [47,53]. Experimental microbiota depletion attenuated or abolished some of these beneficial effects, supporting a functional contribution of the gut microbiota rather than a merely associative relationship [53]. More recent evidence from a stress-induced model further suggests that microbiota-dependent metabolic changes may influence microglial polarization and neuroimmune responses [70].

Taken together, these findings indicate that the neuroprotective effects of phlorizin may arise from coordinated modulation of peripheral metabolism, intestinal microbial ecology, and central inflammatory and synaptic pathways. Nevertheless, evidence remains restricted mainly to preclinical models, and its relevance to human neurological or neuropsychiatric disorders remains to be established.

### 6.10. Anti-Aging and Longevity-Associated Effects

Evidence from C. elegans indicates that phlorizin may influence conserved pathways involved in stress resistance, cellular maintenance, and longevity. Activation of DAF-16/FOXO, SKN-1, and autophagy-related mechanisms has been associated with increased resistance to oxidative stress, extended lifespan, and delayed age-associated functional decline [48].

Although these findings are consistent with the antioxidant and cytoprotective properties described in mammalian systems, their translational relevance remains uncertain. In particular, the available longevity evidence derives primarily from an invertebrate model and therefore requires validation in mammalian systems before any conclusions regarding human aging can be drawn.

### 6.11. Ocular Protective Effects

The ocular effects of phlorizin are mainly related to its antioxidant, anti-inflammatory, and anti-angiogenic properties. In retinal pigment epithelial cells, phlorizin attenuated oxidative injury and inflammatory signaling, while experimental choroidal neovascularization models showed reduced vascular leakage and neovascular lesion formation [49]. In diabetic retinopathy models, phlorizin additionally reduced inflammatory and angiogenic responses and improved retinal barrier integrity [52].

These findings identify the eye as another tissue in which the interconnected antioxidant and anti-inflammatory activities of phlorizin may provide protection. However, available evidence remains preclinical, and questions concerning ocular bioavailability, pharmacokinetics, safety, and clinical efficacy remain unresolved.

### 6.12. Effects on Bone Metabolism

Phlorizin has shown to extend different biological activities, as reported in Table 1.

Phlorizin has shown potential osteoprotective effects in cellular and animal models. It promotes osteoblast differentiation and mineralization while reducing osteoclast formation and bone resorption through modulation of osteogenic and bone-remodeling pathways, including Runx2/osteocalcin, RANKL/OPG, and GSK-3β/β-catenin signaling [71].

These observations suggest that phlorizin may influence bone homeostasis through simultaneous effects on bone formation and resorption. Nevertheless, current evidence is limited to preclinical models and does not yet establish its potential for prevention or treatment of osteoporosis in humans.

**Table 1:** Summary of the biological activities, molecular mechanisms, experimental models and outcomes of phlorizin.

Biological/Nutraceutical Activity	Experimental Model(s)	Key Molecular Mechanisms and Signaling Pathways	Primary Biological Effects and Outcomes	Ref.
Antioxidant activity	In vitro cell-free and cellular models; D-galactose-induced aging mice; *Caenorhabditis elegans*; high-fat diet-fed mice; phlorizin-loaded nanofibrous membranes	Nrf2/Keap1; DAF-16/FOXO; modulation of endogenous antioxidant defenses	Increased SOD, CAT, GSH-Px and total antioxidant capacity; reduced ROS and MDA; enhanced resistance to oxidative stress and preservation of tissue integrity	[13,46,47,48,49]
Antidiabetic activity and SGLT inhibition	Experimental diabetic models; diabetic retinopathy model; high-fat/high-fructose diet-fed mice; experimental peritoneal dialysis	Inhibition of SGLT1/SGLT2; α-glucosidase inhibition; NF-κB/HMGB1; NLRP3; VEGF/HIF-1α/Notch1	Increased glucose excretion; attenuation of hyperglycaemia and insulin resistance; reduction in diabetic retinal injury; inhibition of glucose transport	[14,52,53,54]
Anti-inflammatory activity	D-galactose-induced aging mice; retinal cell and CNV models; diabetic wound models; wound-healing biomaterials	TLR4/MyD88/NF-κB; MAPK (p38, ERK, JNK); IL-1β/IκB-α/NF-κB	Reduced TNF-α, IL-1β, IL-6, COX-2 and iNOS; attenuation of tissue inflammation and inflammatory damage	[46,47,49,55]
Metabolic and lipid-regulating effects	High-fat diet-fed mice; high-fat/high-fructose diet-fed mice; type 2 diabetes/NAFLD mouse model	AMPKα; PPARα/CPT1a; PPARγ/SREBP-1c/FASN/ACC1; gut microbiota modulation	Reduced body-weight gain, dyslipidaemia and hepatic steatosis; improved glucose and lipid homeostasis; increased SCFA production and restoration of gut microbiota homeostasis	[13,53,67]
Cardioprotective effects	High-fat diet-fed Sprague-Dawley rats	HK1/NLRP3/Caspase-1; Bax/Bcl-2/caspase-3; oxidative stress and inflammatory pathways	Reduced myocardial oxidative stress, inflammation, apoptosis and pyroptosis; improved biochemical and histological markers of myocardial injury	[69]
Antidiarrheal and spasmolytic potential	Network pharmacology and molecular docking; isolated chicken ileum; bacterial models	TLR4/NF-κB; PI3K/AKT; HIF-1; modulation of intestinal smooth-muscle contraction	Predicted multi-target anti-inflammatory effects; concentration-dependent inhibition of intestinal contractions; antibacterial activity against selected bacterial strains	[60]
Neuroprotection and microbiota–gut–brain axis modulation	D-galactose-induced aging mice; high-fat/high-fructose diet-fed mice; corticosterone-induced depression model	BDNF–ERK–CREB; NF-κB; microglial M1/M2 polarization; gut microbiota and microbial metabolite pathways	Improved learning, memory and synaptic integrity; reduced neuroinflammation; attenuation of depression-like behaviour; microbiota-dependent modulation of neuroimmune pathways	[47,53,70]
Anti-aging and longevity-associated effects	*Caenorhabditis elegans*; D-galactose-induced aging mice	DAF-16/FOXO; SKN-1; autophagy-related pathways; antioxidant and stress-response mechanisms	Increased oxidative-stress resistance and lifespan in *C. elegans*; reduced oxidative and inflammatory damage in aging mice	[47,48]
Ocular protective effects	ARPE-19 cells exposed to UVA or sodium iodate; laser-induced choroidal neovascularization; experimental diabetic retinopathy	MAPK/NF-κB; VEGF; MMP-2/MMP-9; NLRP3; HIF-1α; NF-κB/HMGB1	Reduced ROS, inflammatory and angiogenic mediators; decreased vascular leakage and CNV; preservation of retinal structure and barrier integrity	[49,52]
Antitumor activity	Esophageal cancer cell/tumor models; chemically induced hepatocellular carcinoma in mice	JAK2/STAT3; mitochondrial apoptotic pathways	Inhibition of tumor progression and cancer-associated signalling; reduced tumour development in an experimental HCC model	[62,64]
Hepatoprotective effects	Type 2 diabetes-associated NAFLD/NASH mouse model	SGLT1/SGLT2 inhibition; metabolic and hepatic lipid-regulatory pathways	Improved glucose homeostasis and hepatic metabolism; reduced hepatic steatosis and pathological alterations associated with metabolic liver disease	[67]
Bone metabolism and osteoprotection	MC3T3-E1 osteoblasts; senescence-accelerated SAMP6 mice	Runx2/osteocalcin; GSK-3β/β-catenin; RANKL/OPG	Enhanced osteoblast differentiation and bone mineralisation; reduced osteoclast formation and bone resorption	[71]
Advanced delivery and wound-healing applications	Chitosan/PVP phlorizin-loaded nanofibers; phlorizin liposome-embedded oxidized sodium alginate/carboxymethyl chitosan hydrogel	Local antioxidant and anti-inflammatory activity; sustained/localized delivery	Enhanced antioxidant and antibacterial properties; improved re-epithelialization, angiogenesis and diabetic/burn wound healing in experimental models	[46,55]
Functional food and protein-based applications	Phlorizin–soy protein conjugate; phlorizin-loaded nanoparticles incorporated into yogurt	Covalent protein–phlorizin interaction; nanoparticle-mediated stabilization and delivery	Modification of protein functionality and development of phlorizin-fortified food formulations with enhanced antioxidant potential	[72,73]

## 7. Nutraceutical Applications, Functional Foods and Advanced Delivery Systems

The broad biological activity of phlorizin has stimulated interest in strategies aimed at improving its stability, bioavailability, and incorporation into food and biomedical formulations. The experimental approaches and principal applications are summarized in Table 1 and Table 2.

### 7.1. Functional Foods and Delivery Systems

Phlorizin has been investigated as a bioactive ingredient for functional foods and dietary formulations. Protein–phlorizin conjugation can modify protein structure and functionality while enhancing selected antioxidant and α-glucosidase inhibitory properties [72]. Nanotechnology-based approaches have also been explored to improve the stability and delivery of phlorizin. In particular, protein-based nanoparticles have enabled its incorporation into food matrices such as yogurt while improving selected physicochemical and antioxidant properties [73].

These approaches illustrate the potential of protein conjugation and nanoencapsulation to overcome some limitations associated with phlorizin stability and bioavailability. However, the available evidence remains largely restricted to formulation, physicochemical, and in vitro studies. Consequently, these systems should currently be regarded as promising technological strategies rather than clinically validated delivery platforms.

### 7.2. Phlorizin-Based Biomaterials for Wound Healing

The main primary experimental studies analysed the biological activities and emerging applications of phlorizin are summarized in Table 2.

Phlorizin has also been incorporated into engineered biomaterials for wound management. Electrospun chitosan/polyvinylpyrrolidone nanofibers and phlorizin-loaded hydrogel systems combine localized delivery with antioxidant, anti-inflammatory, antimicrobial, and tissue-repair activities [46,55]. Experimental wound models indicate that these formulations can accelerate wound closure and promote tissue regeneration while reducing local inflammatory responses.

The convergence of multiple biological activities within a single delivery platform represents a particularly interesting feature of phlorizin-based biomaterials. Nevertheless, as with food-based delivery systems, the available evidence is predominantly preclinical. Further studies are required to establish long-term safety, pharmacokinetics, optimal formulation characteristics, and clinical effectiveness.

**Table 2:** Main primary experimental studies analysed for the biological activities and emerging applications of phlorizin.

Ref.	Biological Activity/Application	Experimental Model	Main Molecular Mechanisms or Endpoints	Main Findings	Evidence Type
[13]	Antioxidant; metabolic; gut microbiota	C57BL/6J mice fed a high-fat diet for 12 weeks	Redox status, lipid metabolism, gut barrier and microbiota composition; SCFA production	Phlorizin improved hyperlipidaemia and redox status, increased SCFA production, regulated lipid-metabolism and barrier-related genes, and partially restored gut microbiota homeostasis	In vivo
[46]	Antioxidant; antibacterial; wound healing; advanced delivery	Chitosan/PVP/phlorizin nanofibrous membrane; cellular and burn-wound models	Antioxidant activity; inflammatory mediators; tissue repair	Phlorizin-loaded nanofibers showed strong antioxidant and antibacterial properties and promoted re-epithelialization and wound healing while reducing local inflammation	In vitro/in vivo
[47]	Antioxidant; anti-aging; neuroprotection; microbiota–gut–brain axis	D-galactose-induced aging mice	Oxidative stress, apoptosis, inflammatory markers and gut microbiota	Phlorizin improved antioxidant status, reduced apoptosis and inflammatory alterations, preserved hippocampal morphology and partially restored gut microbiota composition	In vivo
[48]	Antioxidant; anti-aging; longevity	*Caenorhabditis elegans*	DAF-16/FOXO, SKN-1, hsp-16.2, sod-3 and autophagy	Phlorizin increased oxidative-stress resistance and lifespan and delayed age-associated functional decline through conserved stress-response and autophagy pathways	In vivo model
[49]	Ocular protection; antioxidant; anti-inflammatory	ARPE-19 cells; laser-induced CNV mouse model	ROS; MAPK/NF-κB; IL-1β, IL-6, TNF-α; VEGF; MMP-2/MMP-9	Phlorizin reduced oxidative stress, inflammatory and angiogenic signalling, vascular leakage and CNV formation	In vitro/in vivo
[52]	Antidiabetic; retinal protection	Streptozotocin-induced diabetic rats	NLRP3, VEGF, HIF-1α, ET-1, Notch1; NF-κB/HMGB1; ZO-1 and occludin	Reduced hyperglycaemia, retinal inflammation and angiogenesis and improved blood–retinal barrier integrity	In vivo
[53]	Metabolic; neuroprotective; microbiota–gut–brain axis	High-fat/high-fructose diet-induced obese mice	Gut microbiota, BDNF–ERK–CREB, NF-κB, synaptic proteins and microbial metabolites	Improved glucose and lipid homeostasis, cognitive performance and synaptic integrity; effects were associated with gut microbiota and microbiota-derived metabolites	In vivo
[54]	SGLT inhibition; glucose transport	Experimental peritoneal dialysis model	SGLT1-mediated glucose transport	Phlorizin reduced glucose transport and glucose absorption across the peritoneal membrane, supporting its use as an experimental SGLT inhibitor	In vivo/ex vivo experimental model
[55]	Advanced delivery; anti-inflammatory; diabetic wound healing	Phlorizin liposome-embedded oxidized sodium alginate/carboxymethyl chitosan system; diabetic wound model	Local antioxidant and anti-inflammatory responses; wound repair	The phlorizin-loaded formulation improved wound healing and provided a localized delivery strategy for diabetic wounds	In vivo
[60]	Antidiarrheal; spasmolytic; antimicrobial	Network pharmacology; molecular docking; isolated chicken ileum; bacterial models	TLR4/NF-κB, PI3K/AKT and HIF-1; intestinal smooth-muscle contraction	Computational analyses identified multiple potential targets; phlorizin inhibited intestinal contractions and showed antibacterial activity against selected bacteria	In silico/ex vivo/in vitro
[62]	Antitumor	Esophageal cancer experimental models	JAK2/STAT3 signalling	Phlorizin isolated from sweet tea inhibited signalling associated with esophageal cancer progression	In vitro/in vivo experimental evidence
[64]	Antitumor	DEN/CCl_4_-induced hepatocellular carcinoma in BALB/c mice	Tumour growth and biochemical/histological parameters	Phlorizin isolated from *Syringodium isoetifolium* reduced tumour development and improved biochemical parameters	In vivo
[67]	Hepatoprotective; metabolic	Type 2 diabetes-associated NAFLD/NASH mouse model	SGLT1/SGLT2 inhibition; hepatic glucose and lipid metabolism	Phlorizin improved glucose homeostasis and hepatic metabolism and reduced hepatic steatosis and NASH-associated alterations	In vivo
[69]	Cardioprotective	High-fat diet-fed Sprague-Dawley rats	HK1/NLRP3/Caspase-1; Bax/Bcl-2/caspase-3; oxidative stress and inflammation	Phlorizin attenuated dyslipidaemia, myocardial oxidative stress, inflammation, apoptosis and pyroptosis and improved myocardial injury markers	In vivo
[70]	Neuroprotection; microbiota–gut–brain axis	Corticosterone-induced depression model in mice	Gut microbiota; cysteine/methionine metabolism; microglial M1/M2 polarization; inflammatory cytokines	Phlorizin reduced depression-like behaviour and hippocampal inflammation and promoted a shift toward an anti-inflammatory microglial phenotype	In vivo/in vitro mechanistic evidence
[71]	Bone metabolism; osteoprotection	MC3T3-E1 osteoblasts; SAMP6 mice	Runx2/osteocalcin; GSK-3β/β-catenin; RANKL/OPG	Phlorizin promoted osteoblastogenesis and mineralisation and reduced osteoclast formation and bone resorption	In vitro/in vivo
[72]	Functional food; protein-based delivery	Soy protein–phlorizin conjugate	Tyrosinase-mediated covalent binding; protein structural and functional changes	Formation of a soy protein–phlorizin conjugate modified protein structure and functionality, supporting its potential use in functional food formulations	In vitro/food technology
[73]	Functional food; advanced delivery	Phlorizin-loaded nanoparticles incorporated into yogurt	Nanoparticle-mediated stabilization and delivery; antioxidant activity	Development of a phlorizin-fortified yogurt formulation with antioxidant potential	Food formulation/in vitro

## 8. Discussion

The literature reviewed in this work supports a broad biological potential of phlorizin, but it also highlights a substantial gap between mechanistic and preclinical evidence and clinically validated applications. A major strength of the available literature is the convergence of findings obtained from different experimental models, which suggests that phlorizin may act through multiple interconnected mechanisms rather than through a single molecular target. However, the level of evidence is highly heterogeneous, and the biological effects described in cellular and animal models should not be directly interpreted as evidence of efficacy in humans.

### 8.1. Biological Activities and Mechanistic Evidence

The best-established molecular property of phlorizin is its ability to inhibit sodium–glucose cotransporters, particularly SGLT1 and SGLT2. This mechanism provides a strong pharmacological basis for its effects on intestinal glucose absorption and renal glucose reabsorption and represents the historical foundation for the development of synthetic SGLT inhibitors [14,23,50,51]. However, the biological profile emerging from more recent studies is considerably broader.

The evidence summarized in this review indicates that phlorizin can modulate several pathways involved in oxidative stress, inflammation, glucose and lipid metabolism, tissue injury, and cellular stress responses. In particular, modulation of the Keap1–Nrf2 axis provides a mechanistic basis for its antioxidant activity, whereas inhibition of TLR4/MyD88/NF-κB and MAPK signaling is repeatedly associated with reduced inflammatory responses. Metabolic studies further suggest effects on AMPK, PPARα, CPT1a, and lipogenic pathways, while studies in *Caenorhabditis elegans* indicate activation of conserved DAF-16/FOXO and SKN-1 stress-response pathways [13,47,48,53]. These mechanisms provide a plausible molecular framework linking the apparently diverse biological activities reported for phlorizin.

Nevertheless, mechanistic plausibility should be distinguished from therapeutic validation. Several reported effects are supported predominantly by in vitro or animal studies, whereas computational and ex vivo findings require additional biological confirmation. For example, the proposed antidiarrheal activity is currently supported by network pharmacology, molecular docking, and isolated intestinal tissue experiments, but appropriate in vivo studies are still required [60]. Similarly, the neuroprotective and microbiota-related effects reported in animal models provide an interesting mechanistic hypothesis concerning the microbiota–gut–brain axis, but they cannot yet establish a comparable effect in humans [47,53,70].

The current evidence therefore supports phlorizin as a biologically active phytochemical with pleiotropic experimental effects, rather than as a clinically established therapeutic agent. This distinction is particularly important when considering nutraceutical and biomedical applications.

### 8.2. Nutraceutical Applications: Potential and Limitations

The nutraceutical potential of phlorizin is closely related to its effects on glucose transport, oxidative stress, inflammation, and metabolic regulation. Phlorizin is already present in some commercial dietary formulations, including preparations combined with compounds such as quercetin, phaseolamin, or soy isoflavones. However, the presence of phlorizin in a commercial supplement should not be interpreted as evidence that the formulation has demonstrated clinical efficacy. The manuscript literature indicates that clinical evidence supporting these applications remains limited [74].

A major translational challenge is the relationship between the molecular activity of phlorizin and its behavior following administration. Limited bioavailability, chemical stability, metabolism, and rapid conversion to phloretin may influence the amount of intact phlorizin reaching target tissues. Consequently, biological activity observed at experimentally selected concentrations cannot necessarily be extrapolated to achievable systemic exposure in humans.

This issue also explains the growing interest in formulation technologies. Rather than simply increasing the amount of phlorizin administered, current research increasingly focuses on improving its stability, incorporation into food matrices, intestinal delivery, and potentially its bioaccessibility and pharmacokinetic profile. These strategies are conceptually relevant, but their success must ultimately be demonstrated through pharmacokinetic and clinical studies rather than through physicochemical characterization alone.

### 8.3. Functional Foods and Food-Matrix Applications

The studies reviewed demonstrate that phlorizin can be incorporated into food matrices and can interact with food proteins in ways that modify both physicochemical and functional properties. Tyrosinase-mediated conjugation with soy protein fractions resulted in structural modifications and increased antioxidant and α-glucosidase inhibitory activities compared with the corresponding unconjugated proteins [72]. These findings support the feasibility of using phlorizin–protein interactions to design multifunctional ingredients.

Similarly, phlorizin-loaded zein and zein/gum arabic nanoparticles have been incorporated into yogurt. The reported formulations showed nanoscale particle dimensions, encapsulation of phlorizin, improved colloidal stability with gum arabic, and increased antioxidant activity in the fortified yogurt [73]. These results provide evidence that food engineering can modify the physicochemical environment of phlorizin and facilitate its incorporation into functional products.

However, the evidence supporting these applications remains predominantly technological and preclinical. Increased antioxidant activity measured by chemical assays, improved water-holding capacity, or enhanced enzyme inhibition in a food matrix does not establish a health benefit in consumers. In particular, no clinical evidence is currently presented in the reviewed literature demonstrating that consumption of phlorizin-fortified foods produces clinically meaningful improvements in glucose homeostasis, metabolic disease, or other human health outcomes.

Thus, functional food research should currently be regarded as an important proof-of-concept stage. Future studies should progress from formulation characterization to gastrointestinal digestion models, intestinal absorption studies, pharmacokinetic evaluation, dose–response investigations, and ultimately controlled human intervention studies.

### 8.4. Advanced Delivery Systems and Biomaterials

Advanced delivery technologies represent another important direction because they directly address some of the limitations associated with phlorizin stability and bioavailability. Protein-based nanoparticles, electrospun nanofibers, liposomal systems, and composite hydrogels have all been investigated in the studies included in this review.

The available evidence is particularly interesting for wound-healing applications. Phlorizin-loaded electrospun chitosan/polyvinylpyrrolidone nanofiber membranes showed antibacterial and antioxidant activity and promoted wound closure, granulation tissue formation, collagen deposition, and angiogenesis in a murine burn model [46]. Similarly, phlorizin-loaded liposomes incorporated into an oxidized sodium alginate/carboxymethyl chitosan hydrogel promoted wound closure in diabetic wound models. The latter system was associated with simultaneous inhibition of the TLR4/MyD88/NF-κB inflammatory pathway and activation of the Nrf2/HO-1 antioxidant pathway [55].

These findings are relevant because they demonstrate that formulation may serve not only as a means of improving phlorizin stability but also as a strategy for delivering the compound locally to damaged tissues. Nevertheless, these systems remain at a preclinical stage. The demonstration of improved wound healing in murine models does not establish efficacy, safety, tolerability, or feasibility in human patients.

Importantly, the literature reviewed here does not provide clinical studies demonstrating the efficacy of phlorizin-based nanofibers, liposomal hydrogels, or other advanced phlorizin delivery systems in humans. Therefore, these technologies should be described as promising experimental approaches rather than clinically validated therapeutic strategies.

### 8.5. Clinical Evidence and Translational Gap

A central finding emerging from this review is the disproportion between the extensive preclinical literature and the limited clinical evidence. Most of the biological activities described for phlorizin have been investigated using biochemical assays, cultured cells, ex vivo tissues, *C. elegans*, or rodent models. These studies provide valuable mechanistic information, but the transition from experimental efficacy to human benefit remains insufficiently documented.

The absence of robust clinical evidence is particularly relevant for claims concerning antidiabetic, cardioprotective, neuroprotective, ocular, anti-aging, and tissue-regenerative effects. Although several mechanisms are biologically plausible and supported by convergent experimental findings, there is currently insufficient evidence to establish phlorizin as an effective clinical intervention for these conditions.

The same distinction applies to nutraceutical and functional food applications. The identification of phlorizin in commercial formulations demonstrates technological and commercial interest but does not constitute clinical validation. Likewise, improvements in antioxidant or enzyme-inhibitory activity observed after incorporation into food matrices provide evidence of functionality but not necessarily of efficacy after consumption.

Future translational research should therefore prioritize standardized pharmacokinetic studies, characterization of phlorizin metabolism and tissue exposure, dose–response relationships, long-term safety, and well-designed controlled clinical trials. Such studies are necessary to determine whether the molecular and preclinical effects summarized in this review can be reproduced at physiologically relevant doses in humans.

### 8.6. Strengths and Limitations of the Present Review

The principal strength of this review is its integration of areas that are frequently considered separately. Phlorizin is discussed not only as a characteristic apple-derived phenolic compound but also in relation to its biosynthesis, horticultural determinants, molecular mechanisms, biological activities, food applications, and advanced delivery systems. This integrated perspective helps connect the plant origin of phlorizin with the technological and biomedical approaches currently being developed to exploit its biological properties.

A further strength is the explicit consideration of the experimental level supporting each proposed activity. By distinguishing computational, biochemical, cellular, ex vivo, animal, and human evidence, the review aims to avoid presenting mechanistic or preclinical observations as equivalent to clinical evidence.

Several limitations should nevertheless be acknowledged. First, the available literature is highly heterogeneous with respect to experimental models, doses, formulations, administration routes, endpoints, and study duration, limiting direct comparison among studies. Second, many reported effects derive from preclinical models, while human evidence remains scarce. Third, the pharmacokinetic behavior, metabolism, bioavailability, and clinically relevant dose range of phlorizin remain insufficiently characterized. Finally, the diversity of proposed applications makes it difficult to establish a single translational pathway from experimental observation to clinical use.

Overall, the evidence supports continued investigation of phlorizin, particularly through studies designed to bridge the gap between molecular mechanisms and human exposure. The next phase of research should focus less on expanding the list of experimental activities and more on validating the most reproducible mechanisms under physiologically relevant conditions and determining whether these effects translate into measurable benefits in humans.

## 9. Conclusions

Phlorizin is a characteristic apple-derived dihydrochalcone whose biological properties extend from the regulation of glucose transport to antioxidant, anti-inflammatory, metabolic, neuroprotective, cardioprotective, ocular, and tissue-reparative effects. The available experimental evidence indicates that these activities involve multiple molecular pathways, including SGLT1/SGLT2 inhibition, Keap1–Nrf2, TLR4/MyD88/NF-κB, MAPK, AMPK-related metabolic signaling, and conserved stress-response pathways.

The literature also supports the feasibility of incorporating phlorizin into functional food matrices and advanced delivery systems, including protein-based nanoparticles, electrospun nanofibers, liposomal systems, and composite hydrogels. However, these applications remain predominantly supported by physicochemical, in vitro, and animal studies. Current evidence is therefore sufficient to support the preclinical potential of phlorizin but remains insufficient to establish broad clinical efficacy of phlorizin-based nutraceuticals, functional foods, or advanced delivery systems in humans.

The main priorities for future research are the definition of pharmacokinetic and dose–response relationships, improvement of bioavailability and stability, standardized evaluation of safety, and well-designed clinical studies. Establishing these parameters will be essential to determine whether the promising biological activities of phlorizin can be translated into reproducible and clinically meaningful health benefits.

## Data Availability

Data are available upon reasonable request. The study dataset is available upon reasonable request and is archived by the corresponding author. The protocol deposited with the Ethics Committee is also available.

## References

[1] Caruso A., Barbarossa A., Tassone A., Ceramella J., Carocci A., Catalano A., Basile G., Fazio A., Iacopetta D., Franchini C. (2020). Pomegranate: Nutraceutical with promising benefits on human health. Appl. Sci..

[2] Iacopetta D., Baldino N., Caruso A., Perri V., Lupi F.R., De Cindio B., Gabriele D., Sinicropi M.S. (2021). Nutraceuticals obtained by SFE-CO2 from cladodes of two *Opuntia ficus-indica* (L.) mill wild in calabria. Appl. Sci..

[3] Rodríguez-Negrete E.V., Morales-González Á., Madrigal-Santillán E.O., Sánchez-Reyes K., Álvarez-González I., Madrigal-Bujaidar E., Valadez-Vega C., Chamorro-Cevallos G., Garcia-Melo L.F., Morales-González J.A. (2024). Phytochemicals and their usefulness in the maintenance of health. Plants.

[4] Bonomo M.G., Cafaro C., Russo D., Calabrone L., Milella L., Saturnino C., Capasso A., Salzano G. (2020). Antimicrobial activity, antioxidant properties and phytochemical screening of *Aesculus hippocastanum* mother tincture against food-borne bacteria. Lett. Drug Des. Disc.

[5] Francomano F., Caruso A., Barbarossa A., Fazio A., La Torre C., Ceramella J., Mallamaci R., Saturnino C., Iacopetta D., Sinicropi M.S. (2019). β-Caryophyllene: A sesquiterpene with countless biological properties. Appl. Sci..

[6] Scalbert A., Williamson G. (2000). Dietary intake and bioavailability of polyphenols. J. Nutr..

[7] Chimento A., Sirianni R., Saturnino C., Caruso A., Sinicropi M.S., Pezzi V. (2016). Resveratrol and its analogs as antitumoral agents for breast cancer treatment. Mini Rev. Med. Chem..

[8] Iacopetta D., Grande F., Caruso A., Mordocco R.A., Plutino M.R., Scrivano L., Ceramella J., Muià N., Saturnino C., Puoci F. (2017). New insights for the use of quercetin analogs in cancer treatment. Fut. Med. Chem..

[9] Williamson G. (2017). The role of polyphenols in modern nutrition. Nutr. Bull..

[10] Vinson J.A., Hao Y., Su X., Zubik L. (1998). Phenol antioxidant quantity and quality in foods: Vegetables. J. Agric. Food Chem..

[11] Haslam E. (1996). Natural polyphenols (vegetable tannins) as drugs: Possible modes of action. J. Nat. Prod. Rev..

[12] Cowan M.M. (1999). Plant products as antimicrobial agents. Clin. Microbiol. Rev..

[13] Liu D., Ji Y., Guo Y., Wang H., Wu Z., Li H., Wang H. (2021). Dietary supplementation of apple phlorizin attenuates the redox state related to gut microbiota homeostasis in C57BL/6J mice fed with a high-fat diet. J. Agric. Food Chem..

[14] Ehrenkranz J.R., Lewis N.G., Kahn C.R., Roth J. (2005). Phlorizin: A review. Diabetes/Metab. Res. Rev..

[15] Gosch C., Halbwirth H., Stich K. (2010). Phloridzin: Biosynthesis, distribution and physiological relevance in plants. Phytochemistry.

[16] Stompor M. (2020). Dihydrochalcones: Methods of Acquisition and Pharmacological Properties—A First Systematic Review. Molecules.

[17] Tian L., Cao J., Zhao T., Liu Y., Khan A., Cheng G. (2021). The Bioavailability, Extraction, Biosynthesis and Distribution of Natural Dihydrochalcone: Phloridzin. Int. J. Mol. Sci..

[18] Shelke V., Kale A., Kulkarni Y.A., Gaikwad A.B. (2024). Phloretin: A comprehensive review of its potential against diabetes and associated complications. J. Pharm. Pharmacol..

[19] Stompor M., Potaniec B., Szumny A., Zieliński P., Żołnierczyk A.K., Anioł M. (2013). Microbial synthesis of dihydrochalcones using *Rhodococcus* and *Gordonia* species. J. Mol. Catal. B Enzym..

[20] Gosch C., Halbwirth H., Kuhn J., Miosic S., Stich K. (2009). Biosynthesis of phloridzin in apple (*Malus domestica* Borkh.). Plant Sci..

[21] Tsao R. (2010). Chemistry and biochemistry of dietary polyphenols. Nutrients.

[22] Niederberger K.E., Tennant D.R., Bellion P. (2020). Dietary intake of phloridzin from natural occurrence in foods. Br. J. Nutr..

[23] Blaschek W. (2017). Natural products as lead compounds for Sodium Glucose Cotransporter (SGLT) inhibitors. Planta Med..

[24] Dare A.P., Yauk Y.-K., Tomes S., McGhie T.K., Rebstock R.S., Cooney J.M., Atkinson R.G. (2017). Silencing a phloretin-specific glycosyltransferase perturbs both general phenylpropanoid biosynthesis and plant development. Plant J..

[25] Gutierrez B.L., Zhong G.Y., Brown S.K. (2018). Genetic diversity of dihydrochalcone content in Malus germplasm. Genet. Resour. Crop Evol..

[26] Jugdé H., Nguy D., Moller I., Cooney J.M., Atkinson R.G. (2008). Isolation and characterization of a novel glycosyltransferase that converts phloretin to phlorizin, a potent antioxidant in apple. FEBS J..

[27] Veberic R., Trobec M., Herbinger K., Hofer M., Grill D., Stampar F. (2005). Phenolic compounds in some apple (Malus domestica Borkh) cultivars of organic and integrated production. J. Sci. Food Agric..

[28] Escarpa A., González M.C. (1998). High-performance liquid chromatography with diode-array detection for the determination of phenolic compounds in peel and pulp from different apple varieties. J. Chromatogr. A.

[29] Rana S., Bhushan S. (2016). Apple phenolics as nutraceuticals: Assessment, analysis and application. J. Food Sci. Technol..

[30] Dhyani P., Sati P., Attri D.C., Sharma E., Soni R., Sharifi-Rad J., Calina D. (2025). Phloridzin as a Nutraceutical for Cancer Prevention and Therapy: A Comprehensive Review of Its Mechanisms, Bioavailability Challenges and Future Applications. Food Sci. Nutr..

[31] Rice-Evans C.A., Miller N.J., Paganga G. (1996). Structure-antioxidant activity relationships of flavonoids and phenolic acids. Free Radic. Biol. Med..

[32] Ni T., Zhang S., Rao J., Zhao J., Huang H., Liu Y., Ding Y., Liu Y., Ma Y., Zhang S. (2024). Phlorizin, an Important Glucoside: Research Progress on Its Biological Activity and Mechanism. Molecules.

[33] Wang Y., Ding Y., Zhao Q., Wu C., Deng C.H., Wang J., Wang Y., Yan Y., Zhai R., Yauk Y.-K. (2023). Dihydrochalcone glycoside biosynthesis in Malus is regulated by two MYB-like transcription factors and is required for seed development. Plant J..

[34] Zhou K., Hu L., Li Y., Chen X., Zhang Z., Liu B., Li P., Gong X., Ma F. (2019). MdUGT88F1-Mediated Phloridzin Biosynthesis Regulates Apple Development and Valsa Canker Resistance. Plant Physiol..

[35] Zhang T., Liang J., Wang P., Xu Y., Wang Y., Wei X., Fan M. (2016). Purification and characterization of a novel phloretin-2′-O-glycosyltransferase favoring phloridzin biosynthesis. Sci. Rep..

[36] Elejalde-Palmett C., Billet K., Lanoue A., De Craene J.-O., Glévarec G., Pichon O., Clastre M., Courdavault V., St-Pierre B., Giglioli-Guivarc’h N. (2019). Genome-wide identification and biochemical characterization of the UGT88F subfamily in Malus x domestica Borkh. Phytochemistry.

[37] Guo X., Deng Z., Yang Y., Zhang B., Zhang Y., Yuan Z., Liu C., Rao Y., Luo Z. (2025). Improving the catalytic activity of glycosyltransferase UGT88F1 for highly efficient biosynthesis of phlorizin through structure-guided engineering. Food Biosci..

[38] Wang L., Li C., Luo K. (2024). Biosynthesis and metabolic engineering of isoflavonoids in model plants and crops: A review. Front. Plant Sci..

[39] Khanizadeh S., Tsao R., Rekika D., Yang R., Charles M.T., Rupasinghe H.P.V. (2008). Polyphenol Composition and Total Antioxidant Capacity of Selected Apple Genotypes for Processing. J. Food Compos. Anal..

[40] Táborský J., Sus J., Lachman J., Šebková B., Adamcová A., Šatínský D. (2021). Dynamics of Phloridzin and Related Compounds in Four Cultivars of Apple Trees during the Vegetation Period. Molecules.

[41] Zhou K., Hu L., Li P., Gong X., Ma F. (2017). Genome-wide identification of glycosyltransferases converting phloretin to phloridzin in Malus species. Plant Sci..

[42] Dare A.P., Tomes S., Cooney J.M., Greenwood D.R., Hellens R.P. (2013). The role of enoyl reductase genes in phloridzin biosynthesis in apple. Plant Physiol. Biochem..

[43] Sarkate A., Saini S.S., Teotia D. (2018). Comparative metabolomics of scab-resistant and susceptible apple cell cultures in response to scab fungus elicitor treatment. Sci. Rep..

[44] Miranda S., Piazza S. (2023). CRISPR/Cas9 genome-editing applied to MdPGT1 in apple results in reduced foliar phloridzin without impacting plant growth. Plant J..

[45] Yan Y., Tian B., Li X., Wang Y., Shi Y., Jiang K., Zhai R., Davies K.M., Ma F., Atkinson R.G. (2026). The polyphenol trade-off: Leaf protection vs fruit attraction in apples driven by the phlorizin glycosyltransferase PGGT1. New Phytol..

[46] Yang Y., Ma S., Li A., Xia G., Li M., Ding C., Sun X., Yan L., Yang M., Zhao T. (2024). Antibacterial and antioxidant phlorizin-loaded nanofiber film effectively promotes the healing of burn wounds. Front. Bioeng. Biotechnol..

[47] Chen H., Dong L., Chen X., Ding C., Hao M., Peng X., Zhang Y., Zhu H., Liu W. (2022). Anti-aging effect of phlorizin on D-galactose–induced aging in mice through antioxidant and anti-inflammatory activity, prevention of apoptosis, and regulation of the gut microbiota. Exp. Gerontol..

[48] Park S., Park S.K. (2022). Anti-oxidant and anti-aging effects of phlorizin are mediated by DAF-16-induced stress response and autophagy in Caenorhabditis elegans. Antioxidants.

[49] Liao Z.Y., Hung C.Y., Hsu Y.J., Liang I.C., Chen Y.C., Sung C.H., Hung C.F. (2025). Phlorizin protects against oxidative stress and inflammation in age-related macular degeneration model. Biomolecules.

[50] Tahrani A.A., Barnett A.H., Bailey C.J. (2013). SGLT inhibitors in management of diabetes. Lancet Diabetes Endocrinol..

[51] Perez Lopez G., Albarran O., Cano Megias M. (2010). Type 2 sodium-glucose cotransporter (SGLT2) inhibitors: From familial renal glucosuria to the treatment of type 2 diabetes mellitus. Nefrologia.

[52] Xie L., Zhang R., Hu C., Li T., Zhang Z.P., Jin M.Y., Gao R., Zhang Z.R., Zheng W., Ju Y. (2025). Experimental validation of the molecular mechanism of phlorizin in the treatment of diabetic retinopathy. Exp. Eye Res..

[53] Zhang S., Wang X., Liu S., Hu C., Meng Y. (2025). Phlorizin ameliorates cognitive and behavioral impairments via the microbiota-gut-brain axis in high-fat and high-fructose diet-induced obese male mice. Brain Behav. Immun..

[54] Martus G., Bergling K., Öberg C.M. (2023). Dual SGLT1/SGLT2 inhibitor phlorizin reduces glucose transport in experimental peritoneal dialysis. Perit. Dial. Int..

[55] Wu X., Ding C., Liu X., Ding Q., Zhang S., Wang Y., Xin C., Wei H., Mao R., Zhang G. (2024). The role of phlorizin liposome-embedded oxidized sodium alginate/carboxymethyl chitosan in diabetic wound healing. Int. J. Biol. Macromol..

[56] Zhao P., Zhang Y., Deng H., Meng Y. (2022). Antibacterial mechanism of apple phloretin on physiological and morphological properties of listeria monocytogenes. Food Sci. Technol..

[57] Bonomo M.G., Russo D., Faraone I., Milella L., Mang S.M., Saturnino C., Sinicropi M.S., Catalano A., Salzano G. (2023). Antimicrobial and antioxidant properties and quantitative screening of phytochemicals of *Fraxinus excelsior* L. and *Eschscholtzia californica* Cham. mother tinctures. Nat. Prod. Res..

[58] Silva N.H.C.S., Garrido-Pascual P., Moreirinha C., Almeida A., Palomares T., Alonso-Varona A., Vilela C., Freire C.S.R. (2020). Multifunctional nanofibrous patches composed of nanocellulose and lysozyme nanofibers for cutaneous wound healing. Int. J. Biol. Macromol..

[59] Sun L., Guo Y., Fu C., Li J., Li Z. (2013). Simultaneous separation and purification of total polyphenols, chlorogenic acid and phlorizin from thinned young apples. Food Chem..

[60] Kushwah R., Jain S., Sahu M., Praisthy L.J., Maurya S., Ansari T., Mishra A.K., Kuznetsov A. (2026). Mechanistic insights into the antidiarrheal action of phlorizin: Modulation of TLR4/NF-κB, PI3K/AKT, and HIF-1 signalling with antispasmodic and antimicrobial effects. Naunyn-Schmiedeberg’s Arch. Pharmacol..

[61] Lin S.C., Chen M.C., Liu S., Callahan V.M., Bracci N.R., Lehman C.W., Dahal B., de la Fuente C.L., Lin C.C., Wang T.T. (2019). Phloretin inhibits zika virus infection by interfering with cellular glucose utilisation. Int. J. Antimicrob. Agents.

[62] Jia Z., Xie Y., Wu H., Wang Z., Li A., Li Z., Yang Z., Zhang Z., Xing Z., Zhang X. (2021). Phlorizin from sweet tea inhibits the progress of esophageal cancer by antagonizing the jak2/stat3 signaling pathway. Oncol. Rep..

[63] Wang L., Li Z.W., Zhang W., Xu R., Gao F., Liu Y.F., Li Y.J. (2014). Synthesis, crystal structure, and biological evaluation of a series of phloretin derivatives. Molecules.

[64] Perumal P., Arthanari U., Sanniyasi E. (2023). Phlorizin isolated from seagrass syringodium isoetifolium inhibits diethylnitrosamine and carbon tetrachloride-induced hepatocellular carcinoma in balb/c mice. S. Afr. J. Bot..

[65] de Oliveira M.R. (2016). Phloretin-induced cytoprotective effects on mammalian cells: A mechanistic view and future directions. Biofactors.

[66] Shin J.W., Kundu J.K., Surh Y.J. (2012). Phloretin inhibits phorbol ester-induced tumor promotion and expression of cyclooxygenase-2 in mouse skin: Extracellular signal-regulated kinase and nuclear factor-kappab as potential targets. J. Med. Food.

[67] David-Silva A., Esteves J.V., Morais M.R.P.T., Freitas H.S., Zorn T.M., Correa-Giannella M.L., Machado U.F. (2020). Dual sglt1/sglt2 inhibitor phlorizin ameliorates non-alcoholic fatty liver disease and hepatic glucose production in type 2 diabetic mice. Diabetes Metab. Syndr. Obes..

[68] Zhang J., Li M., Zhao T., Cao J., Liu Y., Wang Y., Wang Y., Cheng G. (2022). E se tea alleviates acetaminophen-induced liver injury by activating the nrf2 signaling pathway. Food Funct..

[69] Zhang Y., Wang Y., Cheng X., Guo H., Ma D., Song Y., Zhang Y., Wang H., Du H. (2025). Cardioprotective effects of phlorizin on hyperlipidemia-induced myocardial injury: Involvement of suppression in pyroptosis via regulating HK1/NLRP3/Caspase-1 signaling pathway. Appl. Biochem. Biotechnol..

[70] Li L., Chen J., Zhang X., Zhang X., Fu Y., Jiang H., Yin T., Zhang Y., Li X., Hu M. (2025). Phlorizin Alleviates Depression-like Behaviors via Gut Microbiota Reprogramming-Induced Methionine to Inhibit Neuroinflammation in Mice Hippocampus. Pharmaceuticals.

[71] Antika L.D., Lee E.J., Kim Y.H., Kang M.K., Park S.H., Kim D.Y., Oh H., Choi Y.J., Kang Y.H. (2017). Dietary phlorizin enhances osteoblastogenic bone formation through enhancing β-catenin activity via GSK-3β inhibition in a model of senile osteoporosis. J. Nutr. Biochem..

[72] Jia Y., Yan X., Li X., Zhang S., Huang Y., Zhang D., Li Y., Qi B. (2023). Soy protein–phlorizin conjugate prepared by tyrosinase catalysis: Identification of covalent binding sites and alterations in protein structure and functionality. Food Chem..

[73] Zhang J., Song Y., Hu X., Zeng Z., Hu J., Zeng T., Geng F., Wu D. (2023). Nanoparticles loaded with phlorizin fabricate a fortified yogurt with antioxidant potential. Food Biosci..

[74] Li D., Liu F., Wang X., Li X. (2019). Apple polyphenol extract alleviates high-fat-diet-induced hepatic steatosis in male C57BL/6 mice by targeting LKB1/AMPK pathway. J. Agric. Food Chem..

